# Statistical mechanics of biomolecular condensates via cavity methods

**DOI:** 10.1016/j.isci.2023.106300

**Published:** 2023-03-06

**Authors:** Nino Lauber, Ondrej Tichacek, Rudrarup Bose, Christoph Flamm, Luca Leuzzi, T-Y Dora Tang, Kepa Ruiz-Mirazo, Daniele De Martino

**Affiliations:** 1 Biofisika Institute (CSIC, UPV/EHU), Barrio Sarriena s/n. 48940 Leioa, Bizkaia, Spain; 2 Donostia International Physics Center (DIPC), Paseo Manuel de Lardizabal, 4, 20018 Donostia-San Sebastian, Gipuzkoa, Spain; 3 Department of Philosophy (UPV/EHU), Avenida de Tolosa 70, 20018 Donostia–San Sebastian, Gipuzkoa, Spain; 4 Institute of Organic Chemistry and Biochemistry of the Czech Academy of Sciences, Flemingovo náměstí 542/2, 160 00 Praha 6, Czech Republic; 5 Max Planck Institute of Molecular Cell Biology & Genetics, Pfotenhauerstraße 108, 01307 Dresden, Germany; 6 Institute for Theoretical Chemistry, University of Vienna, Vienna, Austria; 7 Department of Physics, Universitá di Roma la Sapienza, Piazzale Aldo Moro 5, 00185 Rome, Italy; 8 Institute of Nanotechnology, Soft and Living Matter Laboratory, Consiglio Nazionale delle Ricerche (CNR-NANOTEC), Piazzale Aldo Moro 5, 00185 Rome, Italy; 9 Ikerbasque Foundation, Alameda Urquijo, 36, 48011 Bilbao, Bizkaia, Spain

**Keywords:** Statistical physics, Statistical mechanics, Molecular interaction

## Abstract

Physical mechanisms of phase separation in living systems play key physiological roles and have recently been the focus of intensive studies. The strongly heterogeneous nature of such phenomena poses difficult modeling challenges that require going beyond mean-field approaches based on postulating a free energy landscape. The pathway we take here is to calculate the partition function starting from microscopic interactions by means of cavity methods, based on a tree approximation for the interaction graph. We illustrate them on the binary case and then apply them successfully to ternary systems, in which simpler one-factor approximations are proved inadequate. We demonstrate the agreement with lattice simulations and contrast our theory with coacervation experiments of associative de-mixing of nucleotides and poly-lysine. Different types of evidence are provided to support cavity methods as ideal tools for modeling biomolecular condensation, giving an optimal balance between the consideration of spatial aspects and fast computational results.

## Highlights

• We apply cavity methods to calculate the free energy of biomolecular condensates
• We reproduce simulations of ternary systems where one-factor approximations fail
• Cavity methods are fast and apt to inverse model coacervation experiments
• We reconstruct the phase diagram of polylysine-nucleotides associative demixing

## Graphical abstract

**Figure:**
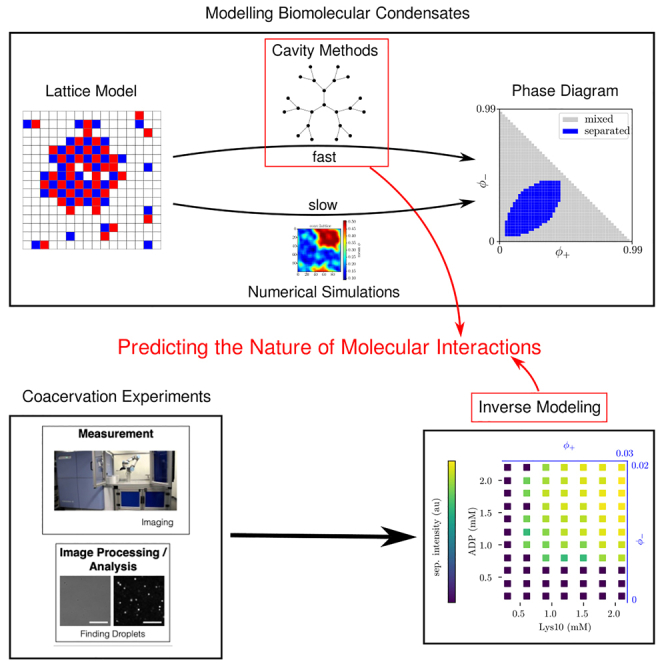

## Introduction

The spatial organization of the components of biological cells is a very important aspect of their physiology^1^ and its nature is eminently physical. For instance, with regard to metabolism, different processes require in principle different environmental conditions and segregation mechanisms to ensure an efficient orchestration of cellular functionalities through compartmentalization. Classical, well-understood examples include oxidative phosphorylation and photosynthesis (performed in specialized organelles, mitochondria, and chloroplasts, respectively^2^). It has been recently proposed that, apart from compartmentalization through lipid membranes, living systems could deal with the problem of creating and controlling microenvironments by means of the physical mechanism of phase separation, where liquid mixtures spatially segregate.^3^ Examples range from ATP concentration in stress granules^4^ to control of gene expression by chromatin condensation^5^^,^^6^ and formation of protein complexes,^7^ while a better-established mechanism is the storage of carbohydrates into starch and/or glycogen,^8^ avoiding potential osmotic imbalance. On the flip side, it is well known that the inadequate formation of biomolecular condensates is the physical correlate of many prion-based pathologies, such as mad cow or Alzheimer’s disease, for instance. Further evidence is accumulating to support that phase separation could play a key role in gene regulation in diseases, including cancer.^9^^,^^10^^,^^11^^,^^12^

Additionally, in the field of origins of life the interest in coacervation has been “rediscovered” in recent years^13^^,^^14^^,^^15^^,^^16^ as a simple and highly plausible compartmentalization mechanism under prebiotic conditions (as it was actually suggested in the early days of the field^17^). It is becoming increasingly apparent, particularly among researchers in the protocell camp, that both physics and chemistry must come together to open the way toward biological complexity, in a scenario in which it is necessary to combine not only a significant diversity of molecular components but also of interactions and transformation processes.^18^

One main difference with respect to classical physical and chemical studies on phase separation is the extremely heterogeneous and complex nature of biological components,^19^ with thousands of different species of microscopic units (that can be complex themselves, such as polymers) even in a relatively simple bacterium such as *E. coli*.^20^ Besides, the specific focus of investigations in life sciences is centered on problems of control, design, and inverse modeling. These aspects spurred the wide use of mean-field approximations for theoretical and computational studies, in particular regarding the extension of the regular solution model.^21^^,^^22^^,^^23^^,^^24^^,^^25^

In the case of polymer solutions, the classical Flory-Huggins (FH) model can be used to describe the segregative de-mixing with the formation of multiple phases, each enriched in one respective polymer.^26^^,^^27^ This model was later extended by Voorn and Overbeek (VO) for solutions of oppositely charged poly-ions (i.e., charged polymers) which usually display associative de-mixing, with the formation of one phase enriched in multiple poly-ions.^28^ Both models are mean-field approximations that build on the interplay between the entropy that drives the mixing of the system and the enthalpy, which results from the interaction energies (derived from van der Waals or ionic forces) between the molecules. As such, they do not explicitly deal with the partition function of the system.

In recent years these mean-field models have been extended successfully to the case of complex coacervation, in particular taking into account the effect of the sequence and focusing on the problem of phase separation of intrinsically disordered proteins, by means of random phase approximation theory.^29^^,^^30^ In this article we will focus on the multi-canonical model defined in ^21^^,^^22^^,^^23^^,^^24^^,^^25^ and its proposed mean-field approximation, namely the regular solution model. We chose to analyze this model for the sake of simplicity, since it is a generalization of the Potts model, which is a minimal model representing an instance of universality class in phase transitions.^31^

One common shortcoming of the aforementioned mean-field approaches is that they tend to neglect spatial correlations by recurring to one-factor approximations, akin to the well-known Curie-Weiss (CW) approximation in magnetic systems.^31^ This is known to lead to difficulties in presence of idiosyncratic, repulsive interactions and frustration, yielding multi-equilibrium. To overcome these difficulties more refined mean-field approximations were developed within the framework of magnetic systems, among which we can find the Bethe-Peierls (BP) approximation,^32^^,^^33^ recently reformulated as cavity methods,^34^ or message passing and belief propagation algorithms.^35^ The latter are considered important standard methods for the statistical physics of spin glasses and disordered systems, with applications that include inference, information theory and resolution of combinatorial optimization problems.^36^

In this work we will apply the BP mean-field technique to describe the self-assembly of biomolecular condensates, focusing more specifically on the problem of reproducing numerical simulations of a general grand-canonical heterogeneous lattice model. The article is organized as follows. First, we will introduce the BP approach and compare it with the regular solution model on a simple standard binary system, providing an analytical formula for the spinodal line that improves quantitatively the match with numerical simulations (in comparison with the classical formula coming from the regular solution model). Then a simple ternary system will be considered, to explore a case where the regular solution model is clearly inadequate (i.e., unable to show even a qualitative agreement with numerical simulations) for the case of mixed repulsive and attractive interactions, known to lead to associative de-mixing. We will demonstrate that the BP approach reproduces much better numerical simulations and provides an immediate method to draw phase diagrams with a semi-quantitative controlled match. This supports the use of BP to classify much more accurately de-mixing phenomena in ternary systems in the interaction coupling space, where we successfully recapitulate the main three modes of phase separation, i.e. i) associative, ii) segregative, and iii) counter-ionic de-mixing. Thus the way is paved for inverse modeling and inference of couplings from experimental results, so our last section will be devoted to the reconstruction of de-mixing phase diagrams from high-throughput data. A relatively simple but well-controlled *in vitro* system with poly-lysine and nucleotides in buffer solution was chosen because it allowed us to explore parameter space more sistematically, it is relevant in the context of prebiotic chemistry and it fulfills our quantitative modeling purposes. Our findings and the potential implications of our work will be finally summarized in a conclusion section.

## Results

As a starting point, we will consider the microscopic coarse-grained multi-component solution model defined e.g in^22^, which can be seen as a particular instance of the generalized Potts model.^37^ The space is discretized into a regular lattice with N sites, where each site-i is in a state $\sigma_{i}=0,1\ldots q$, standing for the presence of a particle of a given type (e.g., solvent or various solutes), q being the number of components. The interaction between two lattice sites $\sigma_{i},\sigma_{j}$ is described by a given function $J\left(\sigma_{i},\sigma_{j}\right)$ (for the usual Potts model $J\left(\sigma_{i},\sigma_{j}\right)=\delta_{\sigma_{i},\sigma_{j}}$) and the number of different kinds of particles is controlled by their chemical potentials $\mu \left(\sigma_{i}\right)$. The Hamiltonian of the system is, therefore:

(Equation 1) $$H\left(\vec{\sigma }\right)=-\sum_{\left\langle i,j\right\rangle }J\left(\sigma_{i},\sigma_{j}\right)+\sum_{i}\mu \left(\sigma_{i}\right)$$

where the first sum runs over all neighboring lattice sites ⟨i,j⟩. In contrast with the regular solution model, we will not postulate a form for the free energy, but rather derive it from microscopic interactions by computing the partition function. This is the fundamental quantity that bridges between the molecular microscopic interactions and the collective macroscopic behavior of the system, alongside with its thermodynamic properties.^3^ Its computation makes it possible to map between the energy as a function of the diverse microscopic configurations and the free energy as a function of macroscopic variables (e.g. concentrations and/or chemical potentials, temperature). Here its expression is (β=1/T is the inverse temperature)

(Equation 2) $$Z=\sum_{\vec{\sigma }}e^{-\beta H\left(\vec{\sigma }\right)}=\sum_{\sigma_{1},\ldots ,\sigma_{N}}e^{\beta \left[\sum_{\left\langle i,j\right\rangle }J\left(\sigma_{i},\sigma_{j}\right)-\sum_{i}\mu \left(\sigma_{i}\right)\right]}$$

**Figure 1:**
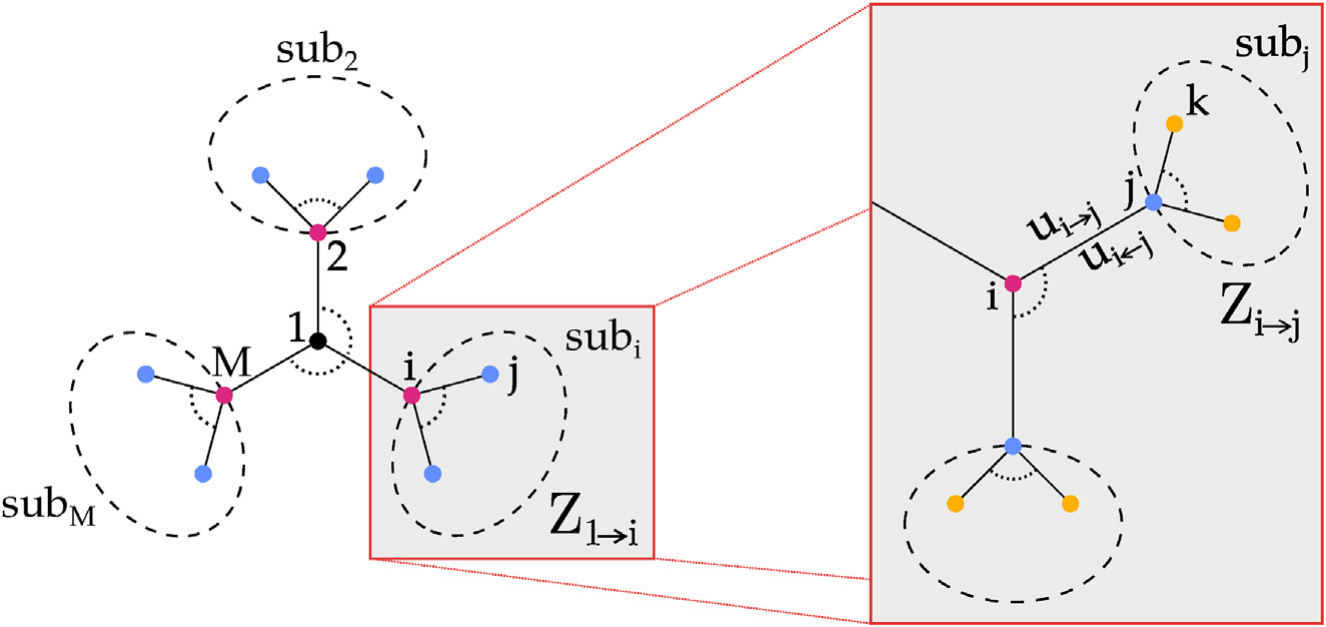
Illustration of the tree-like approximation for the calculation of the partition function Starting from one node, the latter can be decomposed in terms of the conditional partition functions of sub-systems sprouting from neighboring sites and the procedure can be iterated recursively.

We remark that the computation of the partition function is in general a very difficult computational task since an evaluation of the sum on the RHS could require to count objects with involved combinatorics.^36^ For instance, its calculation for the simple Ising model without an external magnetic field corresponds to the enumeration of all closed loop in the underlying graph.^38^ We will compute it by approximating the lattice in terms of a tree-graph branching out from any given site. In this way, the lattice is decomposed into sub-systems that are connected only by the sprouting site. Once the state value of the latter is fixed, the partition function can be factorized in terms of the partition functions of the sub-systems and the procedure can be iterated recursively (see Figure 1).

We end up with the equations

(Equation 3) $$Z_{i\to j}\left(\sigma_{i}\right)=\sum_{\sigma_{j}}e^{\beta \left[J\left(\sigma_{i},\sigma_{j}\right)-\mu \left(\sigma_{j}\right)\right]}\prod_{k\in \partial_{ji}}Z_{j\to k}\left(\sigma_{j}\right)$$

where ∏ stands for the product, $\partial_{ji}$ are all the sites connected to j except from i, and $Z_{i\to j}\left(\sigma_{i}\right)$ is the partition function of the sub-system starting from site-j, given that site-i is fixed to the value $\sigma_{i}$. In general, we have: $Z_{i\to j}\left(\sigma_{i}\right)\neq Z_{j\to i}\left(\sigma_{j}\right)$

### Binary system

For a simple binary phase separation we have q=2 and $\sigma_{i}=0,1$. Fixing $J\left(\sigma_{i},0\right)=\mu \left(0\right)=0$ the cavity equations will be

(Equation 4) $$Z_{i\to j}\left(\sigma_{i}\right)=\prod_{k\in N_{ji}}Z_{j\to k}\left(0\right)+e^{\beta \left[J\left(\sigma_{i},1\right)-\mu \right]}\prod_{k\in N_{ji}}Z_{j\to k}\left(1\right)$$

Parametrizing $Z_{i\to j}\left(\sigma_{i}\right)=A_{i\to j}e^{\beta u_{i\to j}\sigma_{i}}$, it is possible to see that Equation 4 leads to a set of self-consistent equations for the messages $u_{i\to j}$:

(Equation 5) $$u_{i\to j}=\frac{1}{\beta }\log\left(\frac{1+e^{\beta J-\beta \mu +\beta \sum_{k\in N_{ji}}u_{j\to k}}}{1+e^{-\beta \mu +\beta \sum_{k\in N_{ji}}u_{j\to k}}}\right)$$

Considering that the tree-graph is a Caley-Graph with a branching of C=K+1, and assuming homogeneity $u_{i\to j}=u$ (∀i,j) we get for Equation 5:

(Equation 6) $$u=\frac{1}{\beta }\log\left[\frac{1+e^{\beta \left(J-\mu +Ku\right)}}{1+e^{\beta \left(-\mu +Ku\right)}}\right]$$

In addition one can assume that the average site occupation or density (equivalent to the occupation probability of the lattice site by the solute) ⟨σ⟩=φ will verify the following equation (see STAR Methods S.1 for further details):

(Equation 7) $$\begin{array}{cc} \varphi & =\frac{e^{-\beta \mu }\prod_{i\in N_{1}}Z_{1\to i}\left(1\right)}{\prod_{i\in N_{1}}Z_{1\to i}\left(0\right)+e^{-\beta \mu }\prod_{i\in N_{1}}Z_{1\to i}\left(1\right)}\\ & =\frac{e^{-\beta \mu +\beta \left(K+1\right)u}}{1+e^{-\beta \mu +\beta \left(K+1\right)u}} \end{array}$$

**Figure 2:**
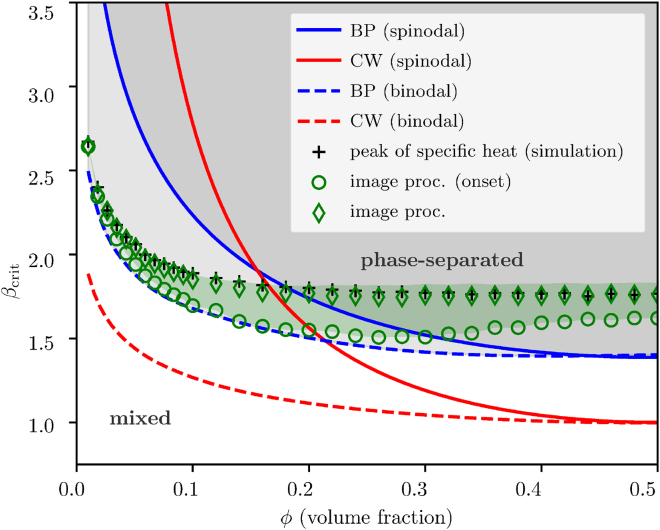
Transition lines for the binary system Comparison of the transition lines (continuous: spinodal, dashed: binodal) of the mean-field regular solution model (Curie-Weiss-like, red curves), the mean-field finite connectivity cavity method (Bethe-Peierls, blue lines) and the numerical simulations on the nearest-neighbor 2D lattice model (points). The black data points are obtained from the peak of the specific heat, whereas the green ones by means of the image processing method described in the text. The green shaded region depicts the transition from a metastable to a phase-separated state as detected by the image processing algorithm (see Figure S3 in the STAR Methods for more details).

These equations express implicitly the state equation φ(μ), from which the phase separation curve (βJ)(φ) can be obtained, in implicit form, by standard thermodynamic stability analysis upon introducing parameter $w=e^{\beta u}$:

(Equation 8) $$\begin{array}{cccc} \frac{\varphi }{1-\varphi } & =\frac{w\left(w-1\right)}{e^{\beta J}-w}\text{,} & w_{1,2} & =\frac{-b\pm \sqrt{b^{2}-4K^{2}e^{\beta J}}}{-2K}\text{,}\\ & & b & =Ke^{\beta J}+K-e^{\beta J}+1 \end{array}$$

where the values $w_{1,2}$ correspond to the two branches of the spinodal line. The above parametric formula can be compared now with the one obtained from the Curie-Weiss model (see STAR Methods S.3 for a derivation)

(Equation 9) $$\beta J=\frac{1}{\left(K+1\right)\left(1-\varphi \right)\varphi }$$

and contrasted with microscopic numerical simulations of the model on a regular square lattice (for which K=3) as illustrated in Figure 2, where we show the binodal lines as well (calculated via Maxwell construction, see STAR Methods S.4). As it can be easily observed, even though the model on a nearest-neighbor lattice is extremely far from being tree-like, numerical simulations are in better quantitative agreement with the predictions of the cavity method, compared with the regular solution equations. An example of the biological application of the phase separation in a binary model to receptor clustering can be found in^39^.

### Ternary system

We next investigated the phase separation between two types of solutes and a solvent. We define configurations as $\sigma_{i}\in \left\{-1,0,+1\right\}$. For the sake of simplicity we assume $J\left(0,\sigma_{i}\right)=J\left(\sigma_{i},0\right)=0\;\mu \left(0\right)=0$ and rename $J\left(-1,-1\right)=J_{--},J\left(+1,+1\right)=J_{++},J\left(-1,+1\right)=J\left(+1,-1\right)=J_{+-}\;\mu \left(-1\right)=\mu_{-},\mu \left(+1\right)=\mu_{+}$. In addition, one can apply the BP method to compute the partition function approximately. In this ternary system, the recursive equations for the partition function of sub-systems along the branches will be

(Equation 10) $$\begin{array}{cc} Z_{i\to j}\left(\sigma_{i}\right) & =e^{\beta \left[J\left(\sigma_{i},-1\right)-\mu_{-}\right]}\prod_{k\in \partial_{ji}}Z_{j\to k}\left(-1\right)\\ & +e^{\beta \left[J\left(\sigma_{i},1\right)-\mu_{+}\right]}\prod_{k\in \partial_{ji}}Z_{j\to k}\left(+1\right)\\ & +\prod_{k\in \partial_{ji}}Z_{j\to k}\left(0\right) \end{array}$$

**Figure 3:**
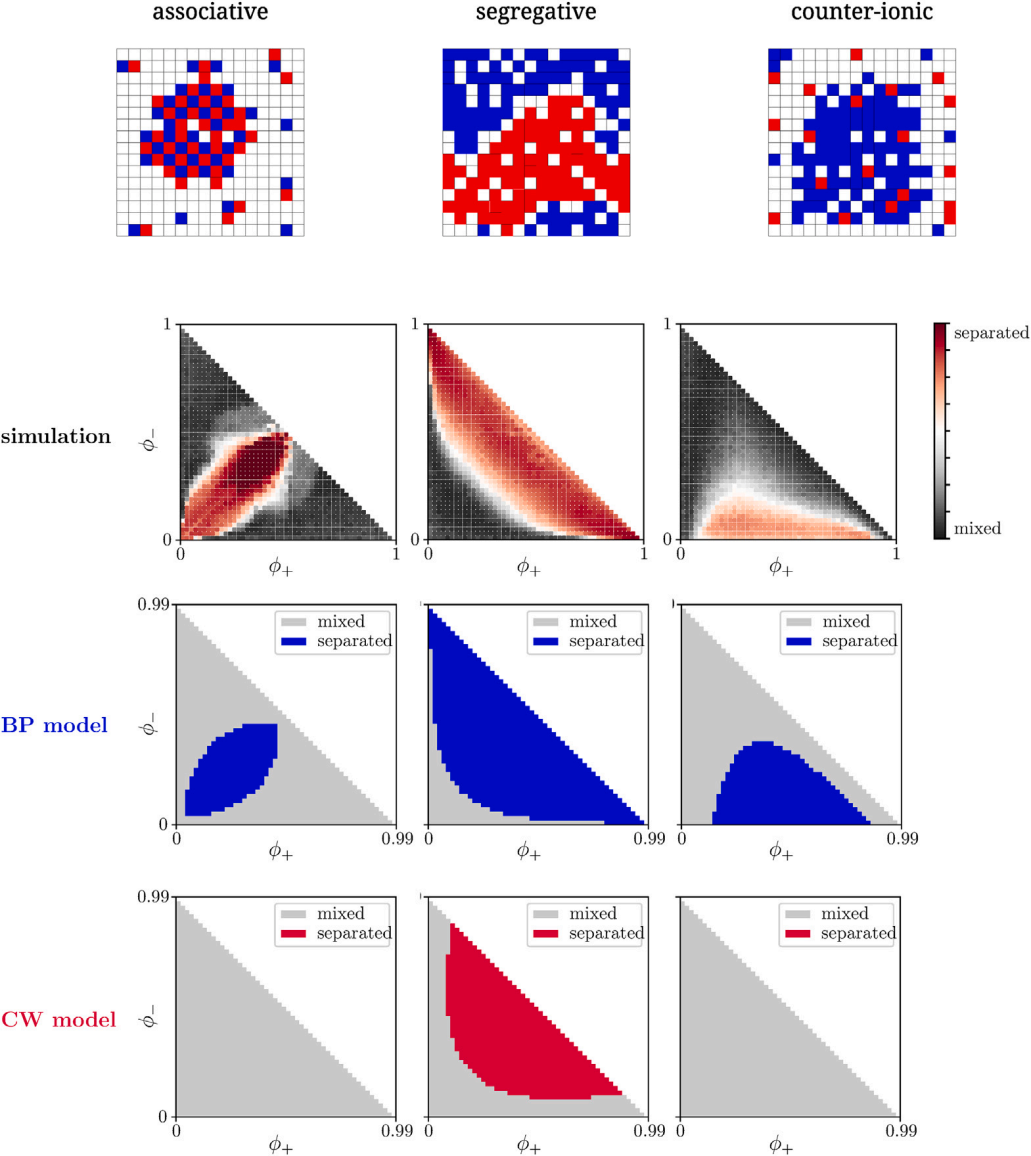
Phase diagrams for the ternary system Comparison of the phase diagrams in the plane of solutes volume fractions $\left(\varphi_{+},\varphi_{-}\right)$ for the ternary system obtained from lattice model simulations (top, obtained from image processing), BP mean-field (center, spinodal), and regular solution mean-field model (bottom, spinodal), for the associative (left $J_{--}=J_{++}=-1,J_{+-}=3$), segregative (middle $J_{--}=J_{++}=1,J_{+-}=-3$) and counter-ionic (right $J_{++}=2,J_{--}=0,J_{+-}=0.5$) de-mixing cases (see text), respectively BP classifies simulation results with 88% accuracy.

Once again these equations can be written in exponential form singling out the dependence on the starting node σ in terms of the so-called message variables. Restricting ourselves to a homogeneous Cayley tree, we assume the homogeneity of the messages $u^{-},u^{+}$, and we obtain the equations

(Equation 11) $$\begin{array}{cc} u^{-} & =\frac{1}{\beta }\log\left[\frac{e^{\beta \left(J_{--}-\mu_{-}+Ku^{-}\right)}+1+e^{\beta \left(J_{+-}-\mu_{+}+Ku^{+}\right)}}{e^{\beta \left(-\mu_{-}+Ku^{-}\right)}+1+e^{\beta \left(-\mu_{+}+Ku^{+}\right)}}\right]\text{,}\\ u^{+} & =\frac{1}{\beta }\log\left[\frac{e^{\beta \left(J_{+-}-\mu_{-}+Ku^{-}\right)}+1+e^{\beta \left(J_{++}-\mu_{+}+Ku^{+}\right)}}{e^{\beta \left(-\mu_{-}+Ku^{-}\right)}+1+e^{\beta \left(-\mu_{+}+Ku^{+}\right)}}\right] \end{array}$$

that together with the average densities

(Equation 12) $$\begin{array}{cc} \varphi_{-} & =\frac{e^{-\beta \mu_{-}+\beta \left(K+1\right)u^{-}}}{e^{-\beta \mu_{-}+\beta \left(K+1\right)u^{-}}+1+e^{-\beta \mu_{+}+\beta \left(K+1\right)u^{+}}}\\ \varphi_{+} & =\frac{e^{-\beta \mu_{+}+\beta \left(K+1\right)u^{+}}}{e^{-\beta \mu_{-}+\beta \left(K+1\right)u^{-}}+1+e^{-\beta \mu_{+}+\beta \left(K+1\right)u^{+}}} \end{array}$$

provide the state equations of the system. The phase diagram can be drawn by checking if the matrix

$$H=\left(\begin{array}{cc} \frac{\partial \mu_{+}}{\partial \varphi_{+}} & \frac{\partial \mu_{+}}{\partial \varphi_{-}}\\ \frac{\partial \mu_{-}}{\partial \varphi_{+}} & \frac{\partial \mu_{-}}{\partial \varphi_{-}} \end{array}\right)$$

is positive definite (by the Routh-Hurwitz criterion detH>0 and trH>0). Results from numerical simulations and mean-field calculations are summarized in Figure 3, where depending on the interaction signs phase separation can be classified into three different kinds: i) associative, ii) segregative, and iii) counter-ionic de-mixing. Those three general types of phase behavior are well accounted for if cavity methods are applied, but more naive or direct mean-field models clearly fail to do so. A strong advantage of mean-field approximations is their low computational cost as compared to an explicit lattice model simulation, considering the fact that the system behavior can be assessed by solving a handful of nonlinear equations. In comparative terms, the time to reconstruct the phase diagrams for the ternary system, shown in Figure 3, differs by 6-7 orders of magnitude when we switch from the lattice model simulations to the resolution of the BP equations (hours vs ms in our implementation). This reduction of computational time enables a full inverse modeling approach to experimental data.

### Modeling experiments

We will consider here the experimental phase diagram of a system of poly-lysine and adenosine-diphosphate (ADP) in buffer solution, as obtained by microscopy imaging. This system, given the residual electrostatic charge of its components, is expected to show typical associative de-mixing behavior, which is not accounted for correctly by the regular mean-field solution model. We considered thus the task of inferring the parameters of the aforementioned ternary solution model that reproduce the experimental phase diagram.

**Figure 4:**
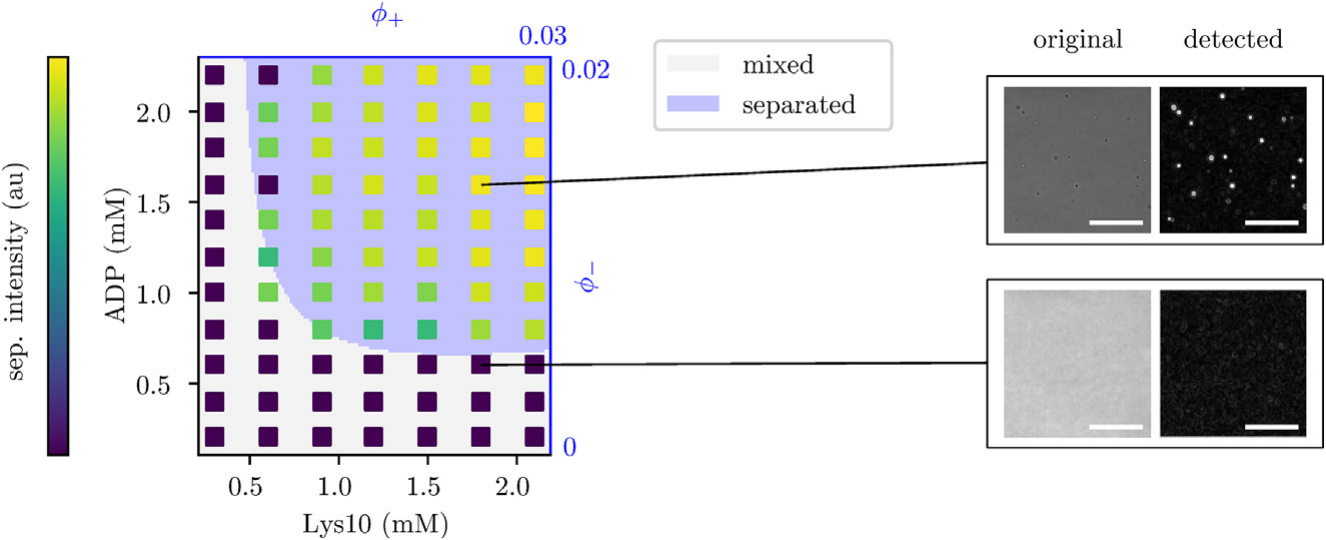
Experimental phase diagram reconstruction Experimental phase diagram in the concentrations plane of a system of poly-lysine and ADP in the salt aqueous solution obtained by microscopic imaging, overlaid by a phase diagram of an inferred ternary system model on a 3D cubic lattice in the volume fractions plane Microscope images of real samples (right) show the formation of the condensate in the form of droplets, scale bars represent 100 μ m. Separation intensity comes from an in-house developed method of automatic image processing of the microscopy images and corresponds to the logarithm of the area of the phase-separated region. The inferred model parameters are $J_{++}=-3.5$, $J_{--}=-2$, $J_{-+}=3.8$. Experimental points are reproduced with 97% accuracy.

This has been formally modeled as a binary classification problem and we implemented an algorithm for parameter inference based on heuristic optimization via differential evolution algorithms.^40^ Results are reported in Figure 4, where we show the experimental phase diagram together with the simulations of the inferred models that are compatible with the associative de-mixing case. Although this provides a quantitative description, a small perturbation in the initial parameter values can lead to an equally well-inferred model, with different parameters. This hints at the presence of many local maxima for the likelihood of model parameters and calls for more refined experiments and/or an inference scheme going beyond simple binary classification. We then performed an inference calculation by constraining the model parameters to be in the region of segregative de-mixing ($J_{--}>0,J_{++}>0,J_{+-}<0$, not shown), obtaining a consistently higher error rate (that is, the fraction of mismatch in binary phase classification, 15%, versus the 3% of the associative case). This shows that our simple setting is able to tell apart the different phase separation mechanisms.

#### Predicted re-entrance in the experimental phase diagram

**Figure 5:**
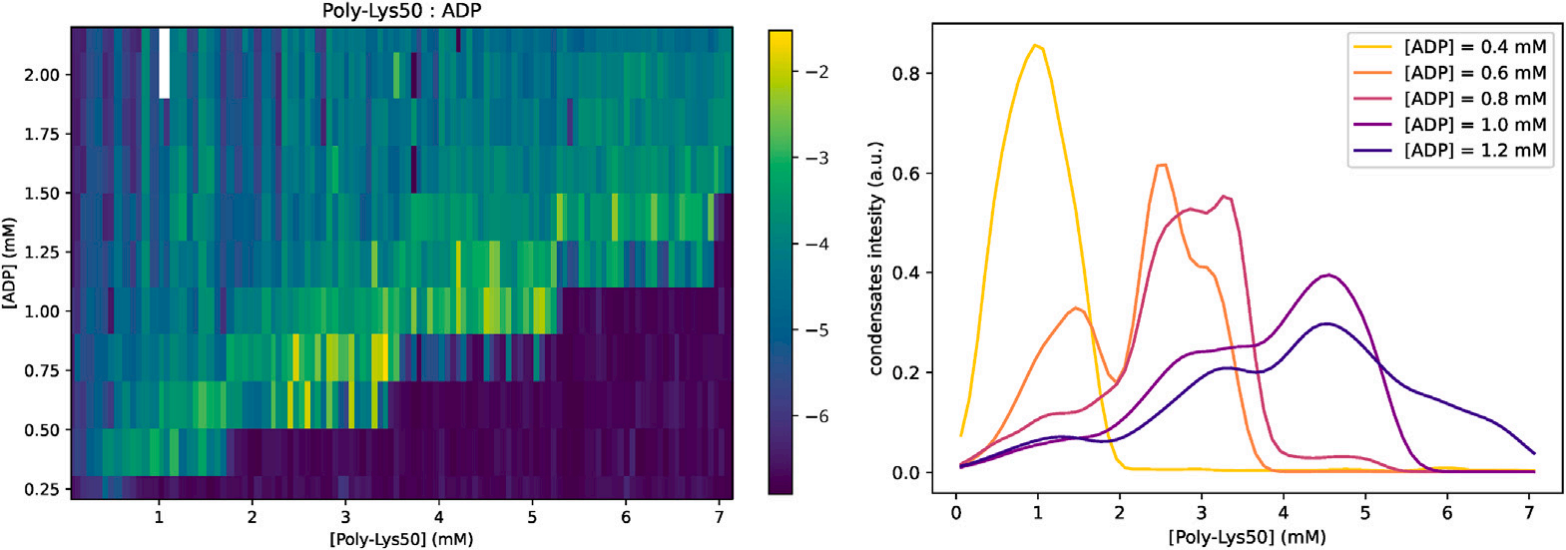
Predicted re-entrance in the phase diagram The concentration of ADP ranged from 0.2 to 2.2 mM and was sampled in 11 steps while the concentration of poly-Lysine ranged from 0.06 to 7.14 mM and was sampled in 112 steps. **Left:** The resulting 11×112 matrix showing the level of phase separation was produced automatically with a custom image-processing method. **Right:** Cuts through the phase diagram at five different constant concentrations of ADP show the ”re-entrance” in the phase diagram, i.e. with increasing concentration of poly-Lysine the phase-separated region appears and then disappears again. The cuts through the phase diagram have been smoothed using a Gaussian kernel-density estimator.

As an additional piece of evidence, let us report here the confirmation of a successful prediction made by our inferred model. Our inferred binding interaction energies predict a form of the phase diagram that falls in the case of associative de-mixing, implying a re-entrance to the symmetric, well-mixed phase for high enough concentrations/volume fractions. Therefore, we performed experiments in that concentration range where such a phenomenon should occur and we found it, in nice agreement with our theoretical prediction (see Figure 5). We remark that the binding energies were inferred in the first part of the phase diagram, apparently showing no sign of re-entrance, so this can be seen as a bona fide prediction or a successful non-trivial generalization.

### Image processing and simulations

**Figure 6:**
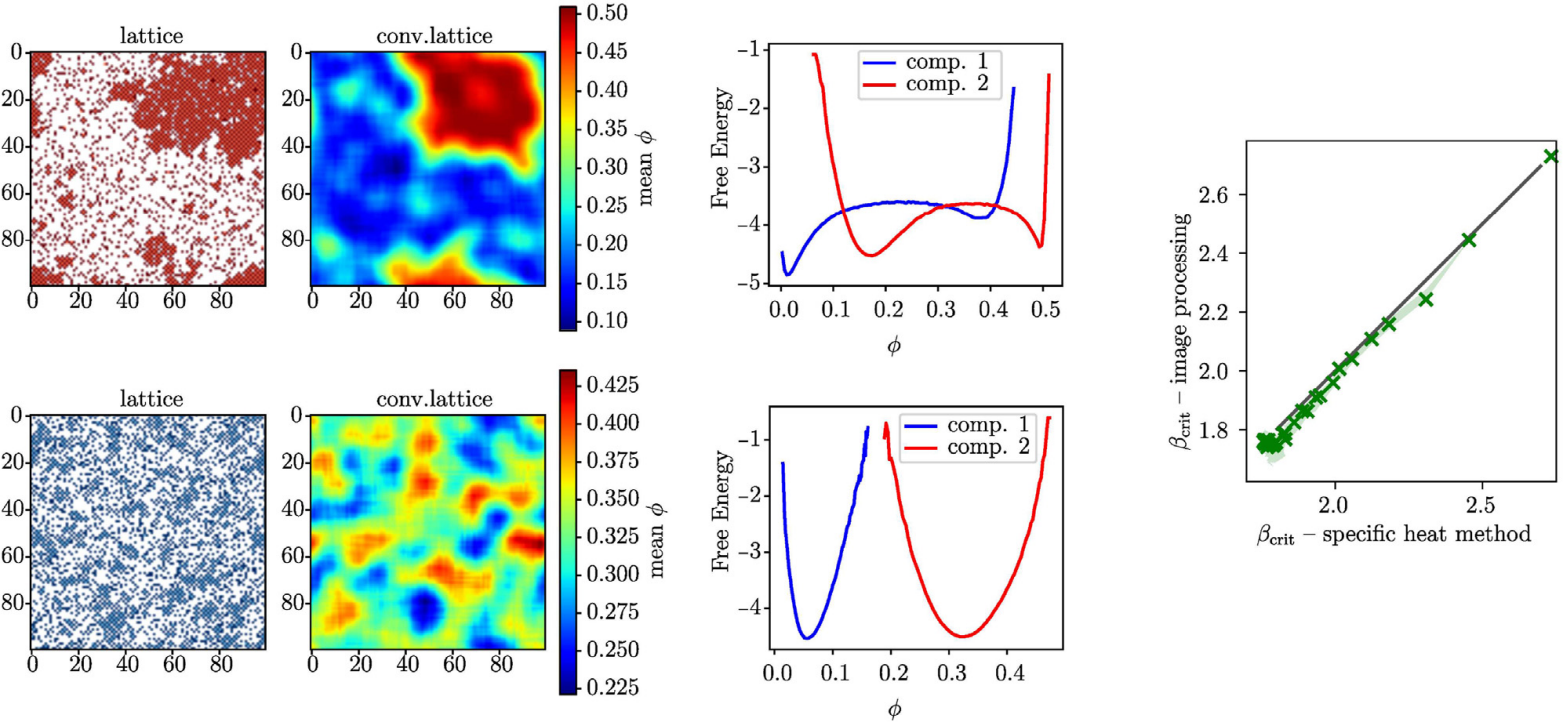
Automated detection of phase separation via image processing Left: lattice model microscopic configuration and its convolution. Center: extracted free energy profile. Two instances of the ternary systems representative of well-mixed and phase-separated behavior respectively. A total of 100 snapshots sampled from the end of the simulation were processed independently. Right: comparison of the inverse critical temperature obtained from specific heat peak computation and image processing automated detection for the binary system.

Numerical simulations were performed via the Monte Carlo Kawasaki scheme,^41^ enforcing fixed volume fraction, and the inverse critical temperature was estimated independently, as the location of the peak of specific heat and the point where the free energy profile changes its concavity (see Figure 6, right). The results of Monte Carlo simulations were analyzed via a heuristic image processing algorithm to identify the occurrence of phase separation. Local particle densities were computed for each component in a lattice snapshot through 2 days convolution with a Gaussian kernel and periodic boundary condition. The logarithm of the distribution of the local density thus obtained was considered via the Gibbs equation as a bona-fide approximation of the free energy of de-mixing. An automated inspection of the number of minima of this reconstructed profile leads to the classification of systems into well-mixed and phase-separated. The procedure is inspired by statistical tests comparing non-parametric distributions and produces a separation confidence score depicted as the color scale in Figure 3. The method is illustrated in Figure 6, left, for two instances of the ternary system that are representative of the well-mixed and phase-separated systems, respectively. The method provided very accurate estimates, as can be seen in Figure 6, right where we show the scattering plot of the inverse critical temperature, at varying volume fractions, for the binary system obtained from the calculation of the peak of the specific heat (see STAR Methods S.5 and S.6).

## Discussion

The study of how biological cells manage or fail to control the spatial/physical conditions of their internal milieu through mechanisms of phase separation is of paramount importance. This should benefit from the wealth of knowledge acquired in the field of the statistical physics of phase transitions in disordered systems, not only in terms of quantitative modeling, but also in data analysis and experiment design. In this article, we have illustrated an application of the mean-field Bethe-Peierls (BP) approximation in the context of heterogeneous phase separation. We have shown that the BP approach quantitatively reconstructs phase diagrams where the standard regular solution model fails even to give a qualitative description: more precisely, in a minimally heterogeneous ternary lattice microscopic model. In particular, the regular solution model^21^^,^^22^^,^^23^^,^^24^^,^^25^ can be seen as coming from an underlying hypothesis of a fully connected graph (see STAR Methods S.2) and this procedure is not guaranteed to provide a reliable approximation in presence of idiosyncratic interactions without appropriate ad-hoc gauge transformations (an elementary example being given by the Ising antiferromagnet^37^).

An explicit derivation of the free energy from the partition function, as we do here with cavity methods, helps in the formulation of an appropriate ansatz for the order parameter form and underlying symmetries. This, apart from being a more adequate theoretical strategy to deal with condensation phenomena, opens a new way for quantitative modeling of experimental data. We actually provided an example reproducing the experimental phase diagram of the associative de-mixing of poly-lysine in the presence of nucleotides. The finding that many models lead to a quantitative description of experimental phase diagrams will deserve further investigation. In this respect, our approach, applied to synthetic data from lattice model simulations could shed light on the right experimental quantities to be measured, leading to well-defined descriptions of the system (i.e., optimal experimental protocols).

In dealing with real data, an interesting ingredient to analyze with our method would be the introduction of inner degeneracy for the basic degrees of freedom, in order to model complex mixtures, since it could also potentially trigger inverse behaviors.^42^ Yet, in order to reproduce the experimental phase diagram we did not need to consider this additional parameter. This could be ascribed to the simplicity of the data being analyzed and to the fact that the interpretation of single lattice points as single molecules is really for illustrative purposes and it should be revised in the light of quantitative comparison with experiments. This is well known for the Ising model, where lattice points are usually not identified with spins of single atoms or molecules but rather with aptly coarse-grained magnetic domains of a given scale.^31^ This aspect is non-trivial and its clarification would touch upon the establishment of a renormalization group approach for these systems, a task that we leave for further investigations.

Although our case study is relatively simple (in comparison with the biomolecular condensates that one can find in a cell’s cytoplasm), it is sufficient for our purposes here, and it is offered also as a relevant example for the origin of life research. Another interesting modification of the model would be to include explicit spatial dependence of the interactions in the form of quenched noise, like in spin glasses, to model in more complex cellular context interactions of the system with other degrees of freedom in a disordered matrix. This could potentially lead to a completely qualitatively different behavior, especially in the case of very heterogeneous idiosyncratic interactions. It is in fact known that the Potts model can be generalized to accommodate for glassy behavior.^43^^,^^44^

One of the caveats of the cavity method consists in its mean-field character, thus limiting its predictive capabilities, in particular for the characterization of the phase transition (e.g. critical exponents^31^). Field theory methods could overcome this aspect and they have been applied successfully in this context recently to model phase separation of intrinsically disordered proteins^45^^,^^46^^,^^47^ see also the recent review.^48^ Apart from that, a promising next step would be to use the BP approach to analyze strongly heterogeneous multi-component systems via replica methods, as it was originally the aim of the regular solution model. In contrast with the latter, BP will be not restricted to the case of mildly attracting interaction matrices. In fact, it could be used to explore any kind of interaction patterns, with idiosyncratic terms, since this approximation showed to be successful in attacking systems with a much more complex free energy landscape, such as spin glasses.

### Limitations of the study

The nature of the proposed cavity method approximation is inherently mean field and thus semi-quantitative. Rigorously, it is known that the free energy calculated in a mean-field approximation (including our approach) is always an upper bound for the true free energy and that mean-field approximations fail to describe quantitatively critical behavior. More refined Monte Carlo numerical simulations or field ftheory-based methods would be needed e.g. for a quantitative assessment of critical exponents. The experimental system we successfully model is arguably rather simple, especially if compared to the complexity of a true cellular environment.

## STAR★Methods

### Key resources table

**Table:** 

REAGENT or RESOURCE	SOURCE	IDENTIFIER
**Chemicals, peptides, and recombinant proteins**		

Poly-L-lysine hydrochloride	Alamanda Polymers	PLKC10 (x=10)
Adenosine 5’-diphosphate sodium salt	Sigma Aldrich	A2754
Trizma base	Sigma Aldrich	T1503

**Software and algorithms**		

KNIME	Open Source	Version 4.3.2 https://www.knime.com/
Automation workflow for mixing 2 components	This paper	
Lattice model numerical simulations	This paper	https://github.com/ondrejtichacek/cavity-phase-sep
Parameter inference optimization procedure	This paper	https://github.com/ondrejtichacek/cavity-phase-sep
Custom image analysis	This paper	https://github.com/ondrejtichacek/cavity-phase-sep
Python 3.9, numpy, scipy, matplotlib, cython	Python Software Foundation	https://www.python.org
Differential Evolution	Storn & Price (1997)	https://doi.org/10.1023/A:1008202821328
Cavity method	Mezard & Montanari (2009)^36^	https://doi.org/10.1093/acprof:oso/9780198570837.001.0001
Kawasaki Monte Carlo	Kawasaki (1966)^41^	https://doi.org/10.1103/PhysRev.145.224
Regular solution model	Jacobs & Fraenkel (2017)^22^	https://doi.org/10.1016/j.bpj.2016.10.043

**Other**		

1536-microwell plate	Greiner bio-one	783096

### Resource availability

#### Lead contact

Further information and requests should be directed to and will be fulfilled by the lead contact, Daniele De Martino (daniele.demartino@ehu.eus).

#### Materials availability

All reagents were obtained from commercial sources and directly used without any further purification. The polymers, poly-lysine of different lengths, were obtained from Alamanda polymers, Inc. Adenosine 5-diphosphate sodium salt (cat. no.: A2754), Adenosine 5-triphosphate disodium salt (cat. no.: A3377), reduced disodium salt hydrate (cat. no.: N8129) were obtained directly from Sigma Aldrich.

### Method details

#### Formulation of the Bethe-Peierls (BP) model

To derive the BP mean field in order to calculate the phase-diagrams, one starts form the Hamiltonian for the system (Equation 1 in the main text). Furthermore, as depicted in Figure 1 in the main text, the lattice is approximated as a tree-graph that branches out from any fixed lattice-site. This allows for the lattice to be decomposed into sub-systems which are only connected through the lattice-site they emerge from, which furthermore means that the Hamiltonian of the system can be decomposed as well:

$$H\left(\vec{\sigma }\right)=-\sum_{i\in \partial_{1}}J\left(\sigma_{1},\sigma_{i}\right)+\mu \left(\sigma_{1}\right)+H_{-1}$$

where $\partial_{1}$ is the neighbourhood of the lattice-site-11 (see again Figure 1 in the main text) and $H_{-1}$ is the Hamiltonian of the system without site-1. Substituting this expression into the partition function (Equation 2 in the main text) results in:

$$Z=\sum_{\vec{\sigma }}e^{-\beta H\left(\vec{\sigma }\right)}=\sum_{\sigma_{1}}\sum_{\sigma_{2},\ldots ,\sigma_{N}}e^{\beta \left[\sum_{i\in \partial_{1}}J\left(\sigma_{1},\sigma_{i}\right)-\mu \left(\sigma_{1}\right)\right]}e^{-\beta H_{-1}}$$

Assuming that there are no interactions between sub-systems, one can write: $H_{-1}=\sum_{i\in \partial_{1}}H_{i}$, where $H_{i}$ is the Hamiltonian of the sub-system emerging from site-i. Thus the partition function can be further decomposed as:

$$\begin{array}{ll} Z & =\sum_{\sigma_{1}}\sum_{\sigma_{2},\ldots ,\sigma_{N}}e^{\beta \sum_{i\in \partial_{1}}J\left(\sigma_{1},\sigma_{i}\right)}e^{-\beta \mu \left(\sigma_{1}\right)}e^{-\beta \sum_{i\in \partial_{1}}H_{i}}\\ & =\sum_{\sigma_{1}}\sum_{\sigma_{2},\ldots ,\sigma_{N}}e^{-\beta \mu \left(\sigma_{1}\right)}e^{\beta \sum_{i\in \partial_{1}}\left[J\left(\sigma_{1},\sigma_{i}\right)-H_{i}\right]}\\ & =\sum_{\sigma_{1}}\sum_{\sigma_{2},\ldots ,\sigma_{N}}e^{-\beta \mu \left(\sigma_{1}\right)}\prod_{i\in \partial_{1}}e^{\beta \left[J\left(\sigma_{1},\sigma_{i}\right)-H_{i}\right]}\\ & =\sum_{\sigma_{1}}e^{-\beta \mu \left(\sigma_{1}\right)}\sum_{\sigma_{2}}\ldots \sum_{\sigma_{N}}e^{\beta \left[J\left(\sigma_{1},\sigma_{2}\right)-H_{2}\right]}\cdot \ldots \cdot e^{\beta \left[J\left(\sigma_{1},\sigma_{M}\right)-H_{M}\right]}\\ & =\sum_{\sigma_{1}}e^{-\beta \mu \left(\sigma_{1}\right)}\sum_{{\text{sub}}_{2}}e^{\beta \left[J\left(\sigma_{1},\sigma_{2}\right)-H_{2}\right]}\cdot \ldots \cdot \sum_{{\text{sub}}_{M}}e^{\beta \left[J\left(\sigma_{1},\sigma_{M}\right)-H_{M}\right]}\\ & =\sum_{\sigma_{1}}e^{-\beta \mu \left(\sigma_{1}\right)}\prod_{i\in \partial_{1}}\underset{Z_{1\to i}\left(\sigma_{1}\right)}{\underset{︸}{\sum_{{\text{sub}}_{i}}e^{\beta \left[J\left(\sigma_{1},\sigma_{i}\right)-H_{i}\right]}}} \end{array}$$

Where ${\text{sub}}_{i}$ is the i-th subsystem, it contains all sites $\sigma_{j}$ that emerge from site $\sigma_{i}$. The partition function is then:

(Equation s1) $$Z=\sum_{\sigma_{1}}e^{-\beta \mu \left(\sigma_{1}\right)}\prod_{i\in \partial_{1}}Z_{1\to i}\left(\sigma_{1}\right)$$

One can further decompose the sub-partition function $Z_{1\to i}\left(\sigma_{1}\right)=\sum_{{\text{sub}}_{i}}e^{\beta J\left(\sigma_{1},\sigma_{i}\right)}e^{-\beta H_{i}}$. As above the Hamiltonian $H_{i}$ of the i-th sub-system can be decomposed into further sub-systems (see again Figure 1 of the main text):

$$H_{i}=-\sum_{j\in \partial_{i\setminus 1}}J\left(\sigma_{i},\sigma_{j}\right)+\mu \left(\sigma_{i}\right)+H_{-i}$$

where $\partial_{i\setminus 1}$ is the neighbourhood of the lattice-site-i without lattice-site-1 (and it’s connected subs-systems) and $H_{-i}$ is the Hamiltonian of the sub-system emerging from lattice-site-i. As above, one can assume that there no interactions between sub-systems, thus one can write $H_{-i}=\sum_{j\in \partial_{i\setminus 1}}H_{j}$. The sub-partition function can then be further decomposed as:

$$\begin{array}{ll} Z_{1\to i}\left(\sigma_{1}\right) & =\sum_{\sigma_{i}}\sum_{{\text{sub}}_{i}\setminus \sigma_{i}}e^{\beta J\left(\sigma_{1},\sigma_{i}\right)}e^{\beta \sum_{j\in \partial_{i\setminus 1}}J\left(\sigma_{i},\sigma_{j}\right)}e^{-\beta \mu \left(\sigma_{i}\right)}e^{-\beta \sum_{j\in \partial_{i\setminus 1}}H_{j}}\\ & =\sum_{\sigma_{i}}e^{\beta \left[J\left(\sigma_{1},\sigma_{i}\right)-\mu \left(\sigma_{i}\right)\right]}\sum_{{\text{sub}}_{i}\setminus \sigma_{i}}e^{\beta \sum_{j\in \partial_{i\setminus 1}}\left[J\left(\sigma_{i},\sigma_{j}\right)-H_{j}\right]}\\ & =\sum_{\sigma_{i}}e^{\beta \left[J\left(\sigma_{1},\sigma_{i}\right)-\mu \left(\sigma_{i}\right)\right]}\sum_{{\text{sub}}_{i}\setminus \sigma_{i}}\prod_{j\in \partial_{i\setminus 1}}e^{\beta \left[J\left(\sigma_{i},\sigma_{j}\right)-H_{j}\right]}\\ & =\sum_{\sigma_{i}}e^{\beta \left[J\left(\sigma_{1},\sigma_{i}\right)-\mu \left(\sigma_{i}\right)\right]}\sum_{{\text{sub}}_{i}\setminus \sigma_{i}}\prod_{j\in \partial_{i\setminus 1}}e^{\beta \left[J\left(\sigma_{i},\sigma_{j}\right)-H_{j}\right]}\\ & =\sum_{\sigma_{i}}e^{\beta \left[J\left(\sigma_{1},\sigma_{i}\right)-\mu \left(\sigma_{i}\right)\right]}\prod_{j\in \partial_{i\setminus 1}}\underset{Z_{i\to j}\left(\sigma_{i}\right)}{\underset{︸}{\sum_{{\text{sub}}_{j}}e^{\beta \left[J\left(\sigma_{i},\sigma_{j}\right)-H_{j}\right]}}} \end{array}$$

In other words, the sub-partition function for the sub-systems emerging from site-1 is:

$$Z_{1\to i}\left(\sigma_{1}\right)=\sum_{\sigma_{i}}e^{\beta \left[J\left(\sigma_{1},\sigma_{i}\right)-\mu \left(\sigma_{i}\right)\right]}\prod_{j\in \partial_{i\setminus 1}}Z_{i\to j}\left(\sigma_{i}\right)$$

This can be generalized to:

(Equation s2) $$Z_{i\to j}\left(\sigma_{i}\right)=\sum_{\sigma_{j}}e^{\beta \left[J\left(\sigma_{i},\sigma_{j}\right)-\mu \left(\sigma_{j}\right)\right]}\prod_{k\in \partial_{j\setminus i}}Z_{j\to k}\left(\sigma_{j}\right)$$

which is Equation 3 in the main text. In other words $Z_{i\to j}\left(\sigma_{i}\right)$ is the sub-partition function of the sub-system starting from lattice-site-j given that lattice-site-j itself starts from lattice-site-i (i.e. lattice-site-i is fixed). In general we have: $Z_{i\to j}\left(\sigma_{i}\right)\neq Z_{j\to i}\left(\sigma_{j}\right)$. In the following sections this partition function will be solved for the special cases of a binary- and a ternary solution.

##### Binary system

As described in the main text, for a binary solution, i.e. q=2 and $\sigma_{i}=0,1$, one can fix $J\left(\sigma_{i},0\right)=\mu \left(0\right)=0$, as well as J(1,1)=J,μ(1)=μ. Putting this into Equation s1 one can simplify the partition function:

$$\begin{array}{ll} Z & =\sum_{\sigma_{1}=0,1}e^{-\beta \mu \left(\sigma_{1}\right)}\prod_{i\in \partial_{1}}Z_{1\to i}\left(\sigma_{1}\right)\\ & =\underset{=1}{\underset{︸}{e^{-\beta \mu \left(0\right)}}}\prod_{i\in \partial_{1}}Z_{1\to i}\left(0\right)+e^{-\beta \overset{=\mu }{\overset{︷}{\mu \left(1\right)}}}\prod_{i\in \partial_{1}}Z_{1\to i}\left(1\right) \end{array}$$

Therefore the partition function for a the binary solution is:

(Equation s3) $$Z=\prod_{i\in \partial_{1}}Z_{1\to i}\left(0\right)+e^{-\beta \mu }\prod_{i\in \partial_{1}}Z_{1\to i}\left(1\right)$$

In a similar way one can simplify Equation s2:

$$\begin{array}{ll} Z_{i\to j}\left(\sigma_{i}\right) & =\sum_{\sigma_{j}=0,1}e^{\beta \left[J\left(\sigma_{i},\sigma_{j}\right)-\mu \left(\sigma_{j}\right)\right]}\prod_{k\in \partial_{j\setminus i}}Z_{j\to k}\left(\sigma_{j}\right)\\ & =\underset{=1}{\underset{︸}{e^{\beta \left[J\left(\sigma_{i},0\right)-\mu \left(0\right)\right]}}}\prod_{k\in \partial_{j\setminus i}}Z_{j\to k}\left(0\right)+e^{\beta \left[J\left(\sigma_{i},1\right)-\overset{=\mu }{\overset{︷}{\mu \left(1\right)}}\right]}\prod_{k\in \partial_{j\setminus i}}Z_{j\to k}\left(1\right) \end{array}$$

which gives the sub-partition function as:

(Equation s4) $$Z_{i\to j}\left(\sigma_{i}\right)=\prod_{k\in \partial_{j\setminus i}}Z_{j\to k}\left(0\right)+e^{\beta \left[J\left(\sigma_{i},1\right)-\mu \right]}\prod_{k\in \partial_{j\setminus i}}Z_{j\to k}\left(1\right)$$

which is Equation 4 in the main text. As further described in the main text, one use the following parametrization: $Z_{i\to j}\left(\sigma_{i}\right)=A_{i\to j}e^{\beta u_{i\to j}\sigma_{i}}$. One can apply this to the RHS and the LHS of Equation s4 to get:

$$\begin{array}{ll} A_{i\to j}e^{\beta u_{i\to j}\sigma_{i}} & =\prod_{k\in \partial_{j\setminus i}}A_{j\to k}e^{\beta u_{j\to k}\cdot 0}+e^{\beta \left[J\left(\sigma_{i},1\right)-\mu \right]}\prod_{k\in \partial_{j\setminus i}}A_{j\to k}e^{\beta u_{j\to k}\cdot 1}\\ & =\prod_{k\in \partial_{j\setminus i}}A_{j\to k}\left(1+e^{\beta \left[J\left(\sigma_{i},1\right)-\mu \right]}e^{\beta \sum_{k\in \partial_{j\setminus i}}u_{j\to k}}\right) \end{array}$$

Next one can take the fraction $Z_{i\to j}\left(1\right)/Z_{i\to j}\left(0\right)$:

$$\begin{array}{ll} \frac{A_{i\to j}e^{\beta u_{i\to j}}}{A_{i\to j}} & =\frac{\prod_{k\in \partial_{j\setminus i}}A_{j\to k}\left(1+e^{\beta \left[J\left(1,1\right)-\mu +\sum_{k\in \partial_{j\setminus i}}u_{j\to k}\right]}\right)}{\prod_{k\in \partial_{j\setminus i}}A_{j\to k}\left(1+e^{\beta \left[J\left(0,1\right)-\mu +\sum_{k\in \partial_{j\setminus i}}u_{j\to k}\right]}\right)}\\ e^{\beta u_{j\to k}} & =\frac{1+e^{\beta \left(J-\mu +\sum_{k\in \partial_{j\setminus i}}u_{j\to k}\right)}}{1+e^{\beta \left(-\mu +\sum_{k\in \partial_{j\setminus i}}u_{j\to k}\right)}} \end{array}$$

Taking the logarithm on both sides of the above equation results in the following set of self-consistent equations for the messages $u_{i\to j}$:

(Equation s5) $$u_{i\to j}=\frac{1}{\beta }\log\left(\frac{1+e^{\beta J-\beta \mu +\beta \sum_{k\in \partial_{j\setminus i}}u_{j\to k}}}{1+e^{-\beta \mu +\beta \sum_{k\in \partial_{j\setminus i}}u_{j\to k}}}\right)$$

which is Equation 5 from the main text. To further simplify this set of equations, one can assume that the tree-graph is a Caley-Graph with a branching of C=K+1 and that there is homogeneity of the messages, i.e. $u_{i\to j}=u,\forall i,j$. Thus one has $\sum_{k\in \partial_{j\setminus i}}u_{j\to k}=Ku$ as $\left|\partial_{j\setminus i}\right|=K$. One therefore retrieves for Equation s5:

(Equation s6) $$u=\frac{1}{\beta }\log\left[\frac{1+e^{\beta \left(J-\mu +Ku\right)}}{1+e^{\beta \left(-\mu +Ku\right)}}\right]$$

which is Equation 6 from the main text. One can furthermore retrieve the probability $P\left(\sigma_{1}=1\right)$ of a lattice site being in state 1 (i.e. being occupied by a solute particle):

$$\begin{array}{ll} P\left(\sigma_{1}=1\right) & =\frac{e^{-\beta \mu \left(1\right)}\prod_{i\in \partial_{1}}Z_{1\to i}\left(1\right)}{Z}\\ & =\frac{e^{-\beta \mu }\prod_{i\in \partial_{1}}Z_{1\to i}\left(1\right)}{\prod_{i\in \partial_{1}}Z_{1\to i}\left(0\right)+e^{-\beta \mu }\prod_{i\in \partial_{1}}Z_{1\to i}\left(1\right)}\equiv \varphi \end{array}$$

which is essentially the volume-fraction φ solute particles2. Applying the same parametrization from above results in:

$$\begin{array}{ll} \varphi & =\frac{e^{-\beta \mu }\prod_{i\in \partial_{1}}A_{1\to i}e^{\beta u_{1\to i}}}{\prod_{i\in \partial_{1}}A_{1\to i}+e^{-\beta \mu }\prod_{i\in \partial_{1}}A_{1\to i}e^{\beta u_{1\to i}}}\\ & =\frac{e^{-\beta \mu +\beta \sum_{i\in \partial_{1}}u_{1\to i}}}{1+e^{-\beta \mu +\beta \sum_{i\in \partial_{1}}u_{1\to i}}}\\ & =\frac{e^{-\beta \mu +\beta \left(K+1\right)u}}{1+e^{-\beta \mu +\beta \left(K+1\right)u}} \end{array}$$

Again the assumption that the tree-graph is a Caley-Graph, therefore $\left|\partial_{1}\right|=K+1$, as well as the homogeneity $u_{1\to i}=u,\forall i$, was used. This way one retrieves φ is depending on u and μ:

(Equation s7) $$\varphi \left(u,\mu \right)=\frac{e^{-\beta \mu +\beta \left(K+1\right)u}}{1+e^{-\beta \mu +\beta \left(K+1\right)u}}$$

which is Equation 7 from the main text. In order to calculate the phase diagram it is necessary to have both φ and βμ parameterized in the following way:

$$\left\{ \begin{array}{c} \beta \mu \left(u\right)\\ \varphi \left(u\right) \end{array}\right.$$

i.e. φ and βμ as functions of the common parameter u. Starting with βμ(u), one can use Equation s6:

$$\begin{array}{ll} e^{\beta u} & =\frac{1+e^{\beta \left(J-\mu +Ku\right)}}{1+e^{\beta \left(-\mu +Ku\right)}}\\ e^{\beta u}\left[1+e^{\beta \left(-\mu +Ku\right)}\right] & =1+e^{\beta \left(J-\mu +Ku\right)}\\ e^{\beta u}+e^{\beta \left[-\mu +\left(K+1\right)u\right]} & =1+e^{\beta \left(J-\mu +Ku\right)}\\ e^{\beta u}-1 & =e^{-\beta \mu }e^{\beta Ku}\left(e^{\beta J}-e^{\beta u}\right)\\ e^{-\beta \mu } & =\frac{e^{\beta u}-1}{e^{\beta Ku}\left(e^{\beta J}-e^{\beta u}\right)} \end{array}$$

Taking the logarithm on both sides results in:

(Equation s8) $$\beta \mu \left(u\right)=-\log\left(e^{\beta u}-1\right)+\beta Ku+\log\left(e^{\beta J}-e^{\beta u}\right)$$

Similarly for φ(u) one can use Equation s7:

$$\begin{array}{ll} \varphi & =\frac{e^{-\beta \mu +\beta \left(K+1\right)u}}{1+e^{-\beta \mu +\beta \left(K+1\right)u}}\\ \varphi +\varphi e^{-\beta \mu }e^{\beta \left(K+1\right)u} & =e^{-\beta \mu }e^{\beta \left(K+1\right)u}\\ \varphi e^{\beta \mu }+\varphi e^{\beta \left(K+1\right)u} & =e^{\beta \left(K+1\right)u}\\ \varphi e^{\beta \mu } & =\left(1-\varphi \right)e^{\beta \left(K+1\right)u}\\ e^{\beta \left(K+1\right)u} & =\left(\frac{\varphi }{\varphi -1}\right)e^{\beta \mu }\\ \beta u & =\frac{1}{K+1}\left[\log\left(\frac{\varphi }{\varphi -1}\right)+\beta \mu \right] \end{array}$$

substituting βμ with Equation s8 gives:

$$\begin{array}{ll} \beta u & =\frac{1}{K+1}\left[\log\left(\frac{\varphi }{\varphi -1}\right)-\log\left(e^{\beta u}-1\right)+\beta Ku+\log\left(e^{\beta J}-e^{\beta u}\right)\right]\\ \left(K+1\right)\beta u & =\log\left[\frac{\left(\frac{\varphi }{\varphi -1}\right)\left(e^{\beta J}-e^{\beta u}\right)}{e^{\beta u}-1}\right]+\beta Ku\\ e^{K\beta u}e^{\beta u} & =\frac{\left(\frac{\varphi }{\varphi -1}\right)\left(e^{\beta J}-e^{\beta u}\right)}{\left(e^{\beta u}-1\right)}e^{\beta Ku} \end{array}$$

from this one can retrieve:

(Equation s9) $$\left(\frac{\varphi }{\varphi -1}\right)=\frac{e^{\beta u}\left(e^{\beta u}-1\right)}{e^{\beta J}-e^{\beta u}}$$

##### Calculating the spinodal-curve for a binary solution

In order to get the spinodal-curve one usually determines were the first derivative of the chemical potential μ with respect to the volume fraction φ (i.e. second derivative of the free energy F) is zero. As here βμ(u) and φ(u) are functions of the parameter u one needs to take:

$$\frac{\partial \left(\beta \mu \right)}{\partial \varphi }=\frac{\partial \left(\beta \mu \right)}{\partial u}\frac{\partial u}{\partial \varphi }=\frac{\partial \left(\beta \mu \right)}{\partial u}{\left(\frac{\partial \varphi }{\partial u}\right)}^{-1}=\frac{\frac{\partial \left(\beta \mu \right)}{\partial u}}{\frac{\partial \varphi }{\partial u}}=0$$

which is basically describes the Nullkline of the curve. This means that it is sufficient to look at the enumerator being 0, i.e. $\frac{\partial \left(\beta \mu \right)}{\partial u}\to 0$. Therefore one can use Equation s8:

$$\frac{\partial \left(\beta \mu \right)}{\partial u}=-\frac{\beta e^{\beta u}}{e^{\beta u}-1}+\beta K-\frac{\beta e^{\beta u}}{e^{\beta J}-e^{\beta u}}=0$$

substituting $w=e^{\beta u}$ one can solve this equation:

$$\begin{array}{ll} \frac{-\beta w\left(e^{\beta J}-w\right)+\beta K\left(w-1\right)\left(e^{\beta J}-w\right)-\beta w\left(w-1\right)}{\left(w-1\right)\left(e^{\beta J}-w\right)} & =0\\ K\left(w-1\right)\left(e^{\beta J}-1\right) & =w\left(e^{\beta J}-w\right)+w\left(w-1\right)\\ K\left(w-1\right)\left(e^{\beta J}-1\right) & =w\left(e^{\beta J}-1\right)\\ Kwe^{\beta J}-Ke^{\beta J}-Kw^{2}+Kw=we^{\beta J}-w-Kw^{2}+\left(Ke^{\beta J}+k-e^{\beta J}+1\right)w-Ke^{\beta J}\\ & =0 \end{array}$$

The solution to this quadratic equation yields the followng expressions:

$$w_{1,2}=\frac{-\left(Ke^{\beta J}+K-e^{\beta J}+1\right)\pm \sqrt{{\left(Ke^{\beta J}+K-e^{\beta J}+1\right)}^{2}-4K^{2}e^{\beta J}}}{-2K}$$

Collection everything together results in

(Equation s10) $$\begin{array}{ll} \left(\frac{\varphi }{\varphi -1}\right) & =\frac{e^{\beta u}\left(e^{\beta u}-1\right)}{e^{\beta J}-e^{\beta u}}=\frac{w\left(w-1\right)}{e^{\beta J}-w}\\ w_{1,2} & =\frac{-b\pm \sqrt{b^{2}-4K^{2}e^{\beta J}}}{-2K}\\ b & =Ke^{\beta J}+K-e^{\beta J}+1 \end{array}$$

which is Equation 8 from the main text. In conclusion, to get a spinodal-curve one can chose values for βJ in order to get values for $w_{1.2}$. One then substitutes them into Equation s10, to get values for φ. This way one thus has a function φ(βJ), in order to get βJ(φ) one simply takes the inverse ${\left(\varphi (\beta J)\right)}^{-1}=\beta J(\varphi )$.

##### Ternary system

For a ternary solution with $\sigma_{i}\in \left\{-1,0,+1\right\}$ one can assume, like in the binary solution, that $J\left(\sigma_{i},0\right)=\mu \left(0\right)=0$. Furthermore, as described in the main text, one can do the following relabeling: $J\left(-1,-1\right)=J_{--},J\left(+1,+1\right)=J_{++},J\left(-1,+1\right)=J\left(+1,-1\right)=J_{+-},\mu \left(-1\right)=\mu_{-},\mu \left(+1\right)=\mu_{+}$. Putting this into Equation s1 for the partition function gives:

$$\begin{array}{cc} Z & =\sum_{\sigma_{1}=-1,0,+1}e^{-\beta \mu \left(\sigma_{1}\right)}\prod_{i\in \partial_{1}}Z_{1\to i}\left(\sigma_{1}\right)\\ & =e^{-\beta \overset{=\mu_{-}}{\overset{︷}{\mu \left(-1\right)}}}\prod_{i\in \partial_{1}}Z_{1\to i}\left(-1\right)+\underset{=1}{\underset{︸}{e^{-\beta \mu \left(0\right)}}}\prod_{i\in \partial_{1}}Z_{1\to i}\left(0\right)+e^{-\beta \overset{=\mu_{+}}{\overset{︷}{\mu \left(+1\right)}}}\prod_{i\in \partial_{1}}Z_{1\to i}\left(+1\right) \end{array}$$

Thus one retrieves the partition function for ternary solution as:

(Equation s11) $$Z=e^{-\beta \mu_{-}}\prod_{i\in \partial_{1}}Z_{1\to i}\left(-1\right)+\prod_{i\in \partial_{1}}Z_{1\to i}\left(0\right)+e^{-\beta \mu_{+}}\prod_{i\in \partial_{1}}Z_{1\to i}\left(+1\right)$$

In a similar way we one can modify Equation s2:

$$\begin{array}{ll} Z_{i\to j}\left(\sigma_{i}\right) & =\sum_{\sigma_{j}=-1,0,1}e^{\beta \left[J\left(\sigma_{i},\sigma_{j}\right)-\mu \left(\sigma_{j}\right)\right]}\prod_{k\in \partial_{j\setminus i}}Z_{j\to k}\left(\sigma_{j}\right)\\ & =e^{\beta \left[J\left(\sigma_{i},-1\right)-\mu \left(-1\right)\right]}\prod_{k\in \partial_{j\setminus i}}Z_{j\to k}\left(-1\right)+\underset{=1}{\underset{︸}{e^{\beta \left[J\left(\sigma_{i},0\right)-\mu \left(0\right)\right]}}}\prod_{k\in \partial_{j\setminus i}}Z_{j\to k}\left(0\right)\\ & +e^{\beta \left[J\left(\sigma_{i},+1\right)-\mu \left(+1\right)\right]}\prod_{k\in \partial_{j\setminus i}}Z_{j\to k}\left(+1\right) \end{array}$$

Which gives the sub-partition function for the sub-systems in the ternary lattice as:

(Equation s12) $$\begin{array}{cc} Z_{i\to j}\left(\sigma_{i}\right) & =e^{\beta \left[J\left(\sigma_{i},-1\right)-\mu_{-}\right]}\prod_{k\in \partial_{j\setminus i}}Z_{j\to k}\left(-1\right)+\prod_{k\in \partial_{j\setminus i}}Z_{j\to k}\left(0\right)\\ & +e^{\beta \left[J\left(\sigma_{i},1\right)-\mu_{+}\right]}\prod_{k\in \partial_{j\setminus i}}Z_{j\to k}\left(+1\right) \end{array}$$

which is Equation 10 in the main text. Like in the binary solution one can introduce a parametrization: $Z_{i\to j}\left(\sigma_{i}\right)=A_{i\to j}e^{\beta \left(v_{i\to j}\sigma_{i}+w_{i\to j}\sigma_{i}^{2}\right)}$. Applying this to the RHS and the LHS of Equation s12 gives:

$$\begin{array}{c} A_{i\to j}e^{\beta \left(v_{i\to j}\sigma_{i}+w_{i\to j}\sigma_{i}^{2}\right)}=e^{\beta \left[J\left(\sigma_{i},-1\right)-\mu_{-}\right]}\prod_{k\in \partial_{j\setminus i}}A_{j\to k}e^{\beta \overset{=u_{j\to k}^{-}}{\overset{︷}{\left(-v_{j\to k}+w_{j\to k}\right)}}}+\prod_{k\in \partial_{j\setminus i}}A_{j\to k}\\ +e^{\beta \left[J\left(\sigma_{i},+1\right)-\mu_{+}\right]}\prod_{k\in \partial_{j\setminus i}}A_{j\to k}e^{\beta \overset{=u_{j\to k}^{+}}{\overset{︷}{\left(v_{j\to k}+w_{j\to k}\right)}}}\\ =\prod_{k\in \partial_{j\setminus i}}A_{j\to k}(e^{\beta \left[J\left(\sigma_{i},-1\right)-\mu_{-}\right]}e^{\beta \sum_{k\in \partial_{j\setminus i}}u_{j\to k}^{-}}+1\\ +e^{\beta \left[J\left(\sigma_{i},+1\right)-\mu_{+}\right]}e^{\beta \sum_{k\in \partial_{j\setminus i}}u_{j\to k}^{+}}) \end{array}$$

As in the binary solution one can take the fraction $Z_{i\to j}\left(-1\right)/Z_{i\to j}\left(0\right)$:

$$\begin{array}{l} \frac{A_{i\to j}e^{\beta u_{i\to j}^{-}}}{A_{i\to j}}=\frac{\prod_{k\in \partial_{j\setminus i}}A_{j\to k}\left(e^{\beta \left[J_{--}-\mu_{-}+\sum_{k\in \partial_{j\setminus i}}u_{j\to k}^{-}\right]}+1+e^{\beta \left[J_{+-}-\mu_{+}+\sum_{k\in \partial_{j\setminus i}}u_{j\to k}^{+}\right]}\right)}{\prod_{k\in \partial_{j\setminus i}}A_{j\to k}\left(e^{\beta \left[-\mu_{-}+\sum_{k\in \partial_{j\setminus i}}u_{j\to k}^{-}\right]}+1+e^{\beta \left[-\mu_{+}+\sum_{k\in \partial_{j\setminus i}}u_{j\to k}^{+}\right]}\right)}\\ e^{\beta u_{j\to k}^{-}}=\frac{e^{\beta \left(J_{--}-\mu_{-}+\sum_{k\in \partial_{j\setminus i}}u_{j\to k}^{-}\right)}+1+e^{\beta \left(J_{+-}-\mu_{+}+\sum_{k\in \partial_{j\setminus i}}u_{j\to k}^{+}\right)}}{e^{\beta \left(-\mu_{-}+\sum_{k\in \partial_{j\setminus i}}u_{j\to k}^{-}\right)}+1+e^{\beta \left(-\mu_{+}+\sum_{k\in \partial_{j\setminus i}}u_{j\to k}^{+}\right)}} \end{array}$$

The same way one can get for $Z_{i\to j}\left(+1\right)/Z_{i\to j}\left(0\right)$:

$$\begin{array}{l} \frac{A_{i\to j}e^{\beta u_{i\to j}^{+}}}{A_{i\to j}}=\frac{\prod_{k\in \partial_{j\setminus i}}A_{j\to k}\left(e^{\beta \left[J_{+-}-\mu_{-}+\sum_{k\in \partial_{j\setminus i}}u_{j\to k}^{-}\right]}+1+e^{\beta \left[J_{++}-\mu_{+}+\sum_{k\in \partial_{j\setminus i}}u_{j\to k}^{+}\right]}\right)}{\prod_{k\in \partial_{j\setminus i}}A_{j\to k}\left(e^{\beta \left[-\mu_{-}+\sum_{k\in \partial_{j\setminus i}}u_{j\to k}^{-}\right]}+1+e^{\beta \left[-\mu_{+}+\sum_{k\in \partial_{j\setminus i}}u_{j\to k}^{+}\right]}\right)}\\ e^{\beta u_{j\to k}^{+}}=\frac{e^{\beta \left(J_{+-}-\mu_{-}+\sum_{k\in \partial_{j\setminus i}}u_{j\to k}^{-}\right)}+1+e^{\beta \left(J_{++}-\mu_{+}+\sum_{k\in \partial_{j\setminus i}}u_{j\to k}^{+}\right)}}{e^{\beta \left(-\mu_{-}+\sum_{k\in \partial_{j\setminus i}}u_{j\to k}^{-}\right)}+1+e^{\beta \left(-\mu_{+}+\sum_{k\in \partial_{j\setminus i}}u_{j\to k}^{+}\right)}} \end{array}$$

These are again the *messages* for the ternary system. Taking the logarithm on both sides of the above equations one can retrieve get the formulas:

(Equation s13) $$\begin{array}{cc} u_{i\to j}^{-} & =\frac{1}{\beta }\log\left(\frac{e^{\beta J_{--}-\beta \mu_{-}+\beta \sum_{k\in \partial_{j\setminus i}}u_{j\to k}^{-}}+1+e^{\beta J_{+-}-\beta \mu_{+}+\beta \sum_{k\in \partial_{j\setminus i}}u_{j\to k}^{+}}}{e^{-\beta \mu_{-}+\beta \sum_{k\in \partial_{j\setminus i}}u_{j\to k}^{-}}+1+e^{-\beta \mu_{+}+\beta \sum_{k\in \partial_{j\setminus i}}u_{j\to k}^{+}}}\right)\\ u_{i\to j}^{+} & =\frac{1}{\beta }\log\left(\frac{e^{\beta J_{+-}-\beta \mu_{-}+\beta \sum_{k\in \partial_{j\setminus i}}u_{j\to k}^{-}}+1+e^{\beta J_{++}-\beta \mu_{+}+\beta \sum_{k\in \partial_{j\setminus i}}u_{j\to k}^{+}}}{e^{-\beta \mu_{-}+\beta \sum_{k\in \partial_{j\setminus i}}u_{j\to k}^{-}}+1+e^{-\beta \mu_{+}+\beta \sum_{k\in \partial_{j\setminus i}}u_{j\to k}^{+}}}\right) \end{array}$$

Again, one can assume a Caley-Graph with a branching of C=K+1 and $u_{i\to j}^{\pm }=u^{\pm },\forall i,j$. Thus one has $\sum_{k\in \partial_{j\setminus i}}u_{j\to k}^{\pm }=Ku^{\pm }$ as $\left|\partial_{j\setminus i}\right|=K$. Equation s13 then simplifies to:

(Equation s14) $$\begin{array}{cc} u^{-} & =\frac{1}{\beta }\log\left[\frac{e^{\beta \left(J_{--}-\mu_{-}+Ku^{-}\right)}+1+e^{\beta \left(J_{+-}-\mu_{+}+Ku^{+}\right)}}{e^{\beta \left(-\mu_{-}+Ku^{-}\right)}+1+e^{\beta \left(-\mu_{+}+Ku^{+}\right)}}\right]\\ u^{+} & =\frac{1}{\beta }\log\left[\frac{e^{\beta \left(J_{+-}-\mu_{-}+Ku^{-}\right)}+1+e^{\beta \left(J_{++}-\mu_{+}+Ku^{+}\right)}}{e^{\beta \left(-\mu_{-}+Ku^{-}\right)}+1+e^{\beta \left(-\mu_{+}+Ku^{+}\right)}}\right] \end{array}$$

which is Equation 11 in the main text. As in the binary solution on can calculate the probabilities $P\left(\sigma_{1}=-1\right),P\left(\sigma_{1}=+1\right)$ of a lattice site being in the state −1,+1 (i.e. being occupied by a solute particle of the - type or + type):

$$\begin{array}{cc} P\left(\sigma_{1}=-1\right) & =\frac{e^{-\beta \mu_{-}}\prod_{i\in \partial_{1}}Z_{1\to i}\left(-1\right)}{e^{-\beta \mu_{-}}\prod_{i\in \partial_{1}}Z_{1\to i}\left(-1\right)+\prod_{i\in \partial_{1}}Z_{1\to i}\left(0\right)+e^{-\beta \mu_{+}}\prod_{i\in \partial_{1}}Z_{1\to i}\left(+1\right)}\equiv \varphi_{-}\\ P\left(\sigma_{1}=+1\right) & =\frac{e^{-\beta \mu_{+}}\prod_{i\in \partial_{1}}Z_{1\to i}\left(+1\right)}{e^{-\beta \mu_{-}}\prod_{i\in \partial_{1}}Z_{1\to i}\left(-1\right)+\prod_{i\in \partial_{1}}Z_{1\to i}\left(0\right)+e^{-\beta \mu_{+}}\prod_{i\in \partial_{1}}Z_{1\to i}\left(+1\right)}\equiv \varphi_{+} \end{array}$$

Which are essentially the volume-fractions $\varphi_{+},\varphi_{-}$ for the solute particles3. Applying the parametrization from above results in:

$$\varphi_{\pm }=\frac{e^{-\beta \mu_{\pm }}\prod_{i\in \partial_{1}}A_{1i}e^{\beta u_{1\to i}^{\pm }}}{e^{-\beta \mu_{-}}\prod_{i\in \partial_{1}}A_{1i}e^{\beta u_{1\to i}^{-}}+\prod_{i\in \partial_{1}}A_{1i}+e^{-\beta \mu_{+}}\prod_{i\in \partial_{1}}A_{1i}e^{\beta u_{1\to i}^{+}}}=\frac{e^{-\beta \mu_{\pm }}\prod_{i\in \partial_{1}}e^{\beta u_{1\to i}^{\pm }}}{e^{-\beta \mu_{-}}\prod_{i\in \partial_{1}}e^{\beta u_{1\to i}^{-}}+1+e^{-\beta \mu_{+}}\prod_{i\in \partial_{1}}e^{\beta u_{1\to i}^{+}}}=\frac{e^{-\beta \mu_{\pm }}e^{\beta \sum_{i\in \partial_{1}}u_{1\to i}^{\pm }}}{e^{-\beta \mu_{-}}e^{\beta \sum_{i\in \partial_{1}}u_{1\to i}^{-}}+1+e^{-\beta \mu_{+}}e^{\beta \sum_{i\in \partial_{1}}u_{1\to i}^{+}}}=\frac{e^{-\beta \mu_{\pm }}e^{\beta \left(K+1\right)u^{\pm }}}{e^{-\beta \mu_{-}}e^{\beta \left(K+1\right)u^{-}}+1+e^{-\beta \mu_{+}}e^{\beta \left(K+1\right)u^{+}}}\equiv \varphi_{+}$$

assuming again a Caley-Graph with $\left|\partial_{1}\right|=K+1$ and $u_{1\to i}^{\pm }=u^{\pm },\forall i$. This way one retrieves $\varphi_{-},\varphi_{+}$ in a ternary-solution as:

(Equation s15) $$\begin{array}{cc} \varphi_{-} & =\frac{e^{-\beta \mu_{-}+\beta \left(K+1\right)u^{-}}}{e^{-\beta \mu_{-}+\beta \left(K+1\right)u^{-}}+1+e^{-\beta \mu_{+}+\beta \left(K+1\right)u^{+}}}\\ \varphi_{+} & =\frac{e^{-\beta \mu_{+}+\beta \left(K+1\right)u^{+}}}{e^{-\beta \mu_{-}+\beta \left(K+1\right)u^{-}}+1+e^{-\beta \mu_{+}+\beta \left(K+1\right)u^{+}}} \end{array}$$

which is Equation 12 from the main text.

##### Calculating the spinodal-curve for a ternary solution

In order to get the spinodal-curve for a ternary system a small “workaround” is needed. First, one can define the following variable transformation

$$m_{\pm }=-\mu_{\pm }+Cu^{\pm }$$

(recall that for our tree-graph one has C=K+1). One thus gets for Equation s15:

$$\begin{array}{l} \varphi_{\pm }=\frac{e^{\beta m_{\pm }}}{1+e^{\beta m_{-}}+e^{\beta m_{+}}}\\ m_{\pm }=\log\left[\varphi_{\pm }\left(1+e^{\beta m_{-}}+e^{\beta m_{+}}\right)\right] \end{array}$$

Given $\varphi_{\pm }$ one can numerically solve this self-consistent equation to get an estimator for $m_{\pm }$. In a similar way one can use the variable transformation from above on Equation s14 (recall K=C−1):

$$u^{\pm }=\frac{1}{\beta }\log\left[\frac{e^{\beta \left(J_{\pm \pm }+m_{\pm }-u^{\pm }\right)}+e^{\beta \left(J_{+-}+m_{\mp }-u^{\mp }\right)}+1}{e^{\beta \left(m_{-}-u^{-}\right)}+e^{\beta \left(m_{+}-u^{+}\right)}+1}\right]$$

Together with the estimate for $m_{\pm }$ one can again numerically solve this self consistent equation to get an estimate for $u^{\pm }$. Finally one can get $\mu_{\pm }$ from the variable transformation together with the estimates for $m_{\pm }$ and $u^{\pm }$:

$$\mu_{\pm }=-m_{\pm }+Cu^{\pm }$$

This way one retrieves an estimate for the external fields $\mu_{\pm }$ in every point $\left(\varphi_{+},\varphi_{-}\right)$. to get the spinodal-curve one then needs to take the Hessian-matrix:

$$H=\left(\begin{array}{cc} \frac{\partial \mu_{+}}{\partial \varphi_{+}} & \frac{\partial \mu_{+}}{\partial \varphi_{-}}\\ \frac{\partial \mu_{-}}{\partial \varphi_{+}} & \frac{\partial \mu_{-}}{\partial \varphi_{-}} \end{array}\right)$$

(recall that $\mu_{\pm }=\frac{\partial F}{\partial \varphi_{\pm }}$, so $\frac{\partial \mu_{\pm }}{\partial \varphi_{\pm }}=\frac{\partial^{2}F}{\partial \varphi_{\pm }\partial \varphi_{\pm }}$). One can compute the derivatives numerically. With the hessian one can use the property that if tr(H)>0 and det(H)>0 the matrix is positive-definite meaning that the free energy F is concave-upward which means that there is no phase-separation. Thus for all points $\left(\varphi_{+},\varphi_{-}\right)$ where this is is not the case we know that there is phase-separation happening.

#### Formulation of the regular solution model

To derive the mean field equations of the regular solution model one again starts from the Hamiltonian of the system (Equation 1 in the main text). Again the lattice is approximated as a graph, however in contrast to the BP-model one assumes a fully connected graph, rather then a tree. One can furthermore substitute the interaction function $J\left(\sigma_{i},\sigma_{j}\right)$ and the chemical potential $\mu \left(\sigma_{i}\right)$ with a symetric matrix and a vector respectively, i.e.: $J\left(\sigma_{i},\sigma_{j}\right)=J_{\sigma_{i}\sigma_{j}},\mu \left(\sigma_{i}\right)=\mu_{\sigma_{i}}$. As the lattice is now fully connected the pairwise sum runs now over all lattice sites. This means one can rewrite the Hamiltonian to:

$$H\left(\vec{\sigma }\right)=-\frac{1}{2N}\sum_{i=1}^{N}\sum_{j=1}^{N}J_{\sigma_{i}\sigma_{j}}+\sum_{i=1}^{N}\mu_{\sigma_{i}}$$

One can further rewrite $J_{\sigma_{i}\sigma_{j}}$ and $\mu_{\sigma_{i}}$ to:

$$J_{\sigma_{i}\sigma_{j}}=\sum_{k=0}^{q-1}\sum_{l=0}^{q-1}J_{kl}\delta_{k\sigma_{i}}\delta_{l\sigma_{j}},\mu_{\sigma_{i}}=\sum_{k=0}^{q-1}\mu_{k}\delta_{k\sigma_{i}}$$

with 0,…,q−1 being the possible states for $\sigma_{i}$ (q possible states overall4) and $\delta_{k\sigma_{i}}$ being the Kronecker-delta. Substituting this back into the Hamiltonian yields:

$$H\left(\vec{\sigma }\right)=-\frac{1}{2N}\sum_{i,j=1}^{N}\sum_{k,l=0}^{q-1}J_{kl}\delta_{k\sigma_{i}}\delta_{l\sigma_{j}}+\sum_{i=1}^{N}\sum_{k=0}^{q-1}\mu_{k}\delta_{k\sigma_{i}}=\frac{1}{2N}\sum_{k,l=0}^{q-1}J_{kl}\sum_{i,j=1}^{N}\delta_{k\sigma_{i}}\delta_{l\sigma_{j}}+\sum_{k=0}^{q-1}\mu_{k}\sum_{i=1}^{N}\delta_{k\sigma_{i}}=\frac{1}{2N}\sum_{k,l=0}^{q-1}J_{kl}\underset{=N_{k}}{\underset{︸}{\left(\sum_{i=1}^{N}\delta_{k\sigma_{i}}\right)}}\underset{=N_{l}}{\underset{︸}{\left(\sum_{j=1}^{N}\delta_{l\sigma_{j}}\right)}}+\sum_{k=0}^{q-1}\mu_{k}\underset{=N_{k}}{\underset{︸}{\sum_{i=1}^{N}\delta_{k\sigma_{i}}}}$$

with $N_{k}$ being the number lattice sites that are in state k, i.e. the number of type-k molecules. Thus the Hamiltonian of the regular solution model is:

(Equation s16) $$H\left(\vec{\sigma }\right)=-\frac{1}{2N}\sum_{k=0}^{q-1}\sum_{l=0}^{q-1}J_{kl}N_{k}N_{l}+\sum_{k=0}^{q-1}\mu_{k}N_{k}$$

Again one can insert Equation s16 into the partition function (Eq. 2 from the main text) to get:

$$Z=\sum_{\sigma_{1},\ldots ,\sigma_{N}}e^{\beta \left[\frac{1}{2N}\sum_{k,l}J_{kl}N_{k}N_{l}-\sum_{k}\mu_{k}N_{k}\right]}$$

This sum is running over the state vector $\vec{\sigma }=\left(\sigma_{1},\ldots ,\sigma_{i},\ldots ,\sigma_{N}\right)$ (i.e. the vector representing all the state of all the lattice-sites). Having transformed the exponent to be dependent on $N_{k}$ rather than $\sigma_{i}$ one would also want the sum to be running running over the state vector $\vec{N}=\left(N_{0},\ldots ,N_{k},\ldots ,N_{q-1}\right)$ which is the vector representing the amount of lattice-sites that are in a certain sate k. In general a certain state vector $\vec{N}$ can be represented by multiple vectors $\vec{\sigma }$ 5. This multiplicity is accounted for by further introducing a multinomial coefficient in the sum. One can therefore rewrite the partition function to:

$$Z=\sum_{N_{0},\ldots ,N_{q}}\left(\begin{array}{c} N\\ N_{0}\ldots N_{q-1} \end{array}\right)e^{\beta \left[\frac{1}{2N}\sum_{k,l}J_{kl}N_{k}N_{l}-\sum_{k}\mu_{k}N_{k}\right]}$$

One can use Stirling-approximation, i.e. $N!\approx e^{N\left(\log\;N-1\right)}$, to do the following simplification:

$$\left(\begin{array}{c} N\\ N_{0}\ldots N_{q-1} \end{array}\right)=\frac{N!}{N_{0}!\ldots N_{q-1}!}\approx e^{N\left(\log\;N-1\right)-\sum_{k}N_{k}\left(\log\;N_{k}-1\right)}=e^{N\;\log\;N-\sum_{k}N_{k}\log\;N_{k}}$$

Substituting this back into the partition function yields:

$$Z=\sum_{\left\{N_{k}\right\}}\exp\left[\beta \left(\frac{1}{2N}\sum_{k,l=0}^{q-1}J_{kl}N_{k}N_{l}-\sum_{k=0}^{q-1}\mu_{k}N_{k}\right)+N\;\log\;N-\sum_{k=0}^{q-1}N_{k}\log\;N_{k}\right]=\sum_{\left\{N_{k}\right\}}e^{f\left(\vec{N}\right)}$$

which is a partition sum over the state space $\left\{N_{k}\right\}$ with the constraint that $\sum N_{k}=N$. Since $N_{k}$ basically represents the amount of type-k molecules in the system, one has: $\varphi_{k}=\frac{N_{k}}{N}$, with $\varphi_{k}$ being the volume fraction of type-k molecules, with $\sum \varphi_{k}=1$. Thus the state space $\left\{\varphi_{k}\right\}$ is essentially defined by a simplex. One can generalize the above partition sum to an integral over this simplex:

$$Z=\int_{\left\{\varphi_{k}\right\}}e^{-\beta NF\left(\vec{\varphi }\right)}d\vec{\varphi }$$

With $F\left(\vec{\varphi }\right)$ being the free energy of the system. Comparing this integral with the partition sum one can see that $f\left(\vec{N}\right)=-\beta NF\left(\vec{\varphi }\right)$. Therefore one can rewrite the exponent in the partition sum from above in order to get $F\left(\vec{\varphi }\right)$:

$$f\left(\vec{N}\right)=\beta \left(\frac{1}{2N}\sum_{k,l=0}^{q-1}J_{kl}N_{k}N_{l}-\sum_{k=0}^{q-1}\mu_{k}N_{k}\right)+N\;\log\;N-\sum_{k=0}^{q-1}N_{k}\log\;N_{k}=-\beta N\left[-\frac{1}{2N^{2}}\sum_{k,l=0}^{q-1}J_{kl}N_{k}N_{l}+\frac{1}{N}\sum_{k=0}^{q-1}\mu_{k}N_{k}-\frac{1}{\beta }\log\;N+\frac{1}{\beta N}\sum_{k=0}^{q-1}N_{k}\log\;N_{k}\right]=-\beta N\left[-\frac{1}{2}\sum_{k,l=0}^{q-1}J_{kl}\underset{=\phi_{k}}{\underset{︸}{\frac{N_{k}}{N}}}\underset{=\phi_{l}}{\underset{︸}{\frac{N_{l}}{N}}}+\sum_{k=0}^{q-1}\mu_{k}\underset{\phi_{k}}{\underset{︸}{\frac{N_{k}}{N}}}+\frac{1}{\beta }\left(\sum_{k=0}^{q-1}\underset{=\phi_{k}}{\underset{︸}{\frac{N_{k}}{N}}}\log\;N_{k}-\log\;N\right)\right]=-\beta N\left[-\frac{1}{2}\sum_{k,l=0}^{q-1}J_{kl}\phi_{k}\phi_{l}+\sum_{k=0}^{q-1}\mu_{k}\phi_{k}+\frac{1}{\beta }\left(\sum_{k=0}^{q-1}\phi_{k}\log\;N_{k}-\underset{=\sum_{k}\frac{N_{k}}{N}\log\;N}{\underset{︸}{\log\;N}}\right)\right]=-\beta N\left[-\frac{1}{2}\sum_{k,l=0}^{q-1}J_{kl}\phi_{k}\phi_{l}+\sum_{k=0}^{q-1}\mu_{k}\phi_{k}+\frac{1}{\beta }\left(\sum_{k=0}^{q-1}\phi_{k}\underset{=\log\frac{N_{k}}{N}}{\underset{︸}{\left(\log\;N_{k}-\log\;N\right)}}\right)\right]=-\beta N\left[-\frac{1}{2}\sum_{k,l=0}^{q-1}J_{kl}\phi_{k}\phi_{l}+\sum_{k=0}^{q-1}\mu_{k}\phi_{k}+\frac{1}{\beta }\sum_{k=0}^{q-1}\phi_{k}\log\;\phi_{k}\right]$$

Using the relation $f\left(\vec{N}\right)=-\beta NF\left(\vec{\varphi }\right)$ from above one retrieves:

(Equation s17) $$F\left(\vec{\varphi }\right)=-\frac{1}{2}\sum_{k,l=0}^{q-1}J_{kl}\varphi_{k}\varphi_{l}+\sum_{k=0}^{q-1}\mu_{k}\varphi_{k}+\frac{1}{\beta }\sum_{k=0}^{q-1}\varphi_{k}\log\;\varphi_{k}$$

which is the free energy of the regular solution model.

##### Binary system

In a binary system one has $\sigma_{i}=0,1$ i.e. q=2. One can assume for the interactions and the chemical potential: $J_{00}=J_{01}=J_{10}=0,\mu_{0}=0$ as well as $J_{11}=J,\mu_{1}=\mu$. Furthermore for the volume fraction one has: $\varphi_{1}=\varphi$ and $\varphi_{0}=1-\varphi$. This means that Equation s17 simplifies to:

(Equation s18) $$F=-\frac{J\varphi^{2}}{2}+\mu \varphi +\frac{1}{\beta }\left[\varphi \;\log\;\varphi +\left(1-\varphi \right)\log\left(1-\varphi \right)\right]$$

One can get the chemical potential by taking the derivative $\frac{\partial F}{\partial \varphi }=0$:

$$\frac{\partial F}{\partial \varphi }=-J\varphi +\mu +\frac{1}{\beta }\left[\log\;\varphi -\log\left(1-\varphi \right)\right]=0$$

From this one retrieves the chemical potential as:

(Equation s19) βμ=βJφ−logφ+log(1−φ)

##### Ternary system

In a ternary system one has $\sigma_{i}=-1,0,+1$ i.e. q=3. One can assume for the interactions and the chemical potential: $J_{0,0}=J_{0,-1}=J_{-1,0}=J_{0,+1}=J_{+1,0}=0,\mu_{0}=0$ as well as $J_{+1,+1}=J_{++},J_{+1,-1}=J_{-1,+1}=J_{+-},J_{-1,-1}=J_{--},\mu_{-1}=\mu_{-},\mu_{+1}=\mu_{+}$. Furthermore for the volume fraction one has: $\varphi_{-1}=\varphi_{-},\varphi_{+1}=\varphi_{+}$ and $\varphi_{0}=1-\varphi_{-}-\varphi_{+}$. This means that Equation s17 simplifies to:

(Equation s20) $$F=-\frac{J_{--}\phi_{-}^{2}}{2}-J_{+-}\phi_{-}\phi_{+}-\frac{J_{++}\phi_{+}^{2}}{2}+\mu_{-}\phi_{-}+\mu_{+}\phi_{+}+\frac{1}{\beta }\left[\phi_{-}\;\log\;\phi_{-}+\phi_{+}\;\log\;\phi_{+}+\left(1-\phi_{-}-\phi_{+}\right)\log\left(1-\phi_{-}-\phi_{+}\right)\right]$$

One can get the chemical potentials by taking the derivative $\frac{\partial F}{\partial \varphi_{\pm }}=0$:

$$\frac{\partial F}{\partial \varphi_{\pm }}=-J_{\pm \pm }\varphi_{\pm }-J_{+-}\varphi_{\mp }+\mu_{\pm }+\frac{1}{\beta }\left[\log\;\varphi_{\pm }-\log\left(1-\varphi_{-}-\varphi_{+}\right)\right]=0$$

From this one retrieves the chemical potentials as:

(Equation s21) $$\begin{array}{cc} \beta \mu_{-} & =\beta J_{--}\varphi_{-}+\beta J_{+-}\varphi_{+}-\log\;\varphi_{-}+\log\left(1-\varphi_{-}-\varphi_{+}\right)\\ \beta \mu_{+} & =\beta J_{++}\varphi_{+}+\beta J_{+-}\varphi_{-}-\log\;\varphi_{+}+\log\left(1-\varphi_{-}-\varphi_{+}\right) \end{array}$$

#### Retrieving the phase diagrams for a binary-system using the Currie-Weiss (CW) model

The Currie-Weiß (CW) model is another approach in order to calculate the phase-diagram6. Like with the BP-model one starts with the Hamiltonian of the system (Equation 1 in the main text). In a binary-solution with $\sigma_{i}=0,1$ one can substitute $J\left(\sigma_{i},\sigma_{j}\right)=J\sigma_{i}\sigma_{j}$ and $\mu \left(\sigma_{i}\right)=\mu \sigma_{i}$ which results in the Hamiltonian:

(Equation s22) $$H\left(\vec{\sigma }\right)=-J\sum_{\left\langle i,j\right\rangle }\sigma_{i}\sigma_{j}+\mu \sum_{i}\sigma_{i}$$

For the CW-model one make the following approximation:

$$\sigma_{i}=\left\langle \sigma \right\rangle +\delta_{i}$$

with $\delta_{i}\ll 1$. In other words, the state at lattice site-i is approximated with and average value ⟨σ⟩ and a small deviation $\delta_{i}$. This way one can simplify the pairwise-sum from Equation s22:

$$\begin{array}{l} \sum_{\langle i,j\rangle }\sigma_{i}\sigma_{j}=\sum_{\langle i,j\rangle }\left(\langle \sigma \rangle +\delta_{i}\right)\cdot \left(\langle \sigma \rangle +\delta_{j}\right)=\sum_{\langle i,j\rangle }\left[{\langle \sigma \rangle }^{2}+\left(\underset{=\sigma_{i}-\langle \sigma \rangle }{\underset{︸}{\delta_{i}}}+\overset{=\sigma_{j}-\langle \sigma \rangle }{\overset{︷}{\delta_{j}}}\right)\langle \sigma \rangle +\underset{\approx 0}{\underset{︸}{\delta_{i}\delta_{j}}}\right]\approx \sum_{\langle i,j\rangle }\left[{\langle \sigma \rangle }^{2}+\left(\sigma_{i}-\langle \sigma \rangle +\sigma_{j}\langle \sigma \rangle \right)\langle \sigma \rangle \right]=\sum_{\langle i,j\rangle }\left[\sigma_{i}\langle \sigma \rangle +\sigma_{j}\langle \sigma \rangle -{\langle \sigma \rangle }^{2}\right]=\langle \sigma \rangle \sum_{\langle i,j\rangle }\sigma_{i}+\langle \sigma \rangle \sum_{\langle i,j\rangle }\sigma_{j}-{\langle \sigma \rangle }^{2}\sum_{\langle i,j\rangle }1=\langle \sigma \rangle \frac{1}{2}\sum_{i=1}^{N}\sigma_{i}\underset{=k+1}{\underset{︸}{\sum_{j\in N_{i}}1}}+\langle \sigma \rangle \frac{1}{2}\sum_{j=1}^{N}\sigma_{j}\underset{=k+1}{\underset{︸}{\sum_{i\in N_{j}}1}}-{\langle \sigma \rangle }^{2}\frac{1}{2}\underset{N\left(k+1\right)}{\underset{︸}{\sum_{i=1}^{N}\sum_{j\in N_{1}}1}}=\langle \sigma \rangle \frac{k+1}{2}\sum_{i}\sigma_{i}+\langle \sigma \rangle \frac{k+1}{2}\underset{=\sum_{i}\sigma_{i}}{\underset{︸}{\sum_{j}\sigma_{j}}}-{\langle \sigma \rangle }^{2}\frac{N\left(k+1\right)}{2}=\langle \sigma \rangle \left(k+1\right)\sum_{i}\sigma_{i}-{\langle \sigma \rangle }^{2}\frac{N\left(k+1\right)}{2} \end{array}$$

Substituting this back into the Hamiltonian gives:

$$\begin{array}{cc} H & =-J\left[\left\langle \sigma \right\rangle \left(k+1\right)\sum_{i}\sigma_{i}-{\left\langle \sigma \right\rangle }^{2}\frac{N\left(k+1\right)}{2}\right]+\mu \sum_{i}\sigma_{i}\\ & =-J\left\langle \sigma \right\rangle \left(k+1\right)\sum_{i}\sigma_{i}+J{\left\langle \sigma \right\rangle }^{2}\frac{N\left(k+1\right)}{2}+\mu \sum_{i}\sigma_{i} \end{array}$$

This way one can retrieve the Hamiltonian for the CW-model as:

(Equation s23) $$H\left(\vec{\sigma }\right)=J{\left\langle \sigma \right\rangle }^{2}\frac{N\left(k+1\right)}{2}-\left[J\left\langle \sigma \right\rangle \left(k+1\right)-\mu \right]\sum_{i}\sigma_{i}$$

Inserting Equation s23 into the partition function (Equation 2 from the main text) yields:

$$Z=\sum_{\sigma_{1}\ldots \sigma_{N}}e^{-\beta J{\langle \sigma \rangle }^{2}\frac{N\left(k+1\right)}{2}}e^{\beta \left[J\langle \sigma \rangle \left(k+1\right)-\mu \right]\sum_{i}\sigma_{i}}=e^{-\beta J{\langle \sigma \rangle }^{2}\frac{N\left(k+1\right)}{2}}\sum_{\sigma_{1}\ldots \sigma_{N}}\prod_{i}e^{\beta \left[J\langle \sigma \rangle \left(k+1\right)-\mu \right]\sigma_{i}}=e^{-\beta J{\langle \sigma \rangle }^{2}\frac{N\left(k+1\right)}{2}}\sum_{\sigma_{1}}\ldots \sum_{\sigma_{N}}e^{\beta \left[J\langle \sigma \rangle \left(k+1\right)-\mu \right]\sigma_{1}}\cdot \ldots \cdot e^{\beta \left[J\langle \sigma \rangle \left(k+1\right)-\mu \right]\sigma_{N}}=e^{-\beta J{\langle \sigma \rangle }^{2}\frac{N\left(k+1\right)}{2}}\underset{\left(1+e^{\beta \left[J\langle \sigma \rangle \left(k+1\right)-\mu \right]}\right)}{\underset{︸}{\sum_{\sigma_{1}=0,1}e^{\beta \left[J\langle \sigma \rangle \left(k+1\right)-\mu \right]\sigma_{1}}}}\cdot \ldots \cdot \underset{\left(1+e^{\beta \left[J\langle \sigma \rangle \left(k+1\right)-\mu \right]}\right)}{\underset{︸}{\sum_{\sigma_{N}=0,1}e^{\beta \left[J\langle \sigma \rangle \left(k+1\right)-\mu \right]\sigma_{N}}}}$$

Thus resulting in the partition function for the CW-model:

(Equation s24) $$Z=e^{-\beta J{\left\langle \sigma \right\rangle }^{2}\frac{N\left(k+1\right)}{2}}{\left(1+e^{\beta \left[J\left\langle \sigma \right\rangle \left(k+1\right)-\mu \right]}\right)}^{N}$$

Taking the logarithm on both sides yields:

(Equation s25) $$\log\;Z=N\left[\log\left(1+e^{\beta \left[J\left\langle \sigma \right\rangle \left(k+1\right)-\mu \right]}\right)-\beta J{\left\langle \sigma \right\rangle }^{2}\frac{\left(k+1\right)}{2}\right]$$

Looking at the derivative of logZ with respect to βμ for the general expression $Z=\sum_{\sigma_{i}}e^{\beta f_{\mathrm{int}}}e^{-\beta \mu \sum \sigma_{i}}$:

$$\begin{array}{ll} \frac{\partial \log\;Z}{\partial \left(\beta \mu \right)}=\frac{1}{Z}\frac{\partial Z}{\partial \left(\beta \mu \right)} & =-\frac{1}{Z}\sum_{\sigma_{i}}e^{\beta f_{\mathrm{int}}}\sum_{i}\sigma_{i}e^{-\beta \mu \sum \sigma_{i}}\\ & =-\sum_{i}\frac{1}{Z}\sum_{\sigma_{i}}\sigma_{i}e^{\beta f_{\mathrm{int}}}e^{-\beta \mu \sum \sigma_{i}}\\ & =-\sum_{i}\underset{=\left\langle \sigma \right\rangle }{\underset{︸}{\left\langle \sigma_{i}\right\rangle }}=N\left\langle \sigma \right\rangle \end{array}$$

In other words one retrieves the following relation:

$$\left\langle \sigma \right\rangle =-\frac{1}{N}\frac{\partial \log\;Z}{\partial \left(\beta \mu \right)}$$

In general one can substitute ⟨σ⟩=φ, i.e. the average site occupancy corresponds to the volume fraction of the solute. Applying the above relation to the logarithm of Equation s25 gives:

$$\begin{array}{ll} \varphi & =-\frac{1}{N}\frac{\partial }{\partial \left(\beta \mu \right)}N\left[\log\left(1+e^{\beta \left[J\varphi \left(k+1\right)-\mu \right]}\right)-\beta J\varphi^{2}\frac{\left(k+1\right)}{2}\right]\\ & =-\frac{\partial }{\partial \left(\beta \mu \right)}\log\left(1+e^{\beta \left[J\varphi \left(k+1\right)-\mu \right]}\right)\\ & =\frac{e^{\beta \left[J\varphi \left(k+1\right)-\mu \right]}}{1+e^{\beta \left[J\varphi \left(k+1\right)-\mu \right]}} \end{array}$$

Thus one retrieves for the volume fraction φ

$$\varphi \left(\beta \mu \right)=\frac{e^{\beta J\varphi \left(k+1\right)}e^{-\beta \mu }}{1+e^{\beta J\varphi \left(k+1\right)}e^{-\beta \mu }}$$

One can solve this equation now for βμ

$$\begin{array}{ll} \varphi \left(1+e^{\beta J\varphi \left(k+1\right)}e^{-\beta \mu }\right) & =e^{\beta J\varphi \left(k+1\right)}e^{-\beta \mu }\\ \varphi +\varphi e^{\beta J\varphi \left(k+1\right)}e^{-\beta \mu } & =e^{\beta J\varphi \left(k+1\right)}e^{-\beta \mu }\\ e^{\beta J\varphi \left(k+1\right)}e^{-\beta \mu }\left(1-\varphi \right) & =\varphi \\ e^{\beta J\varphi \left(k+1\right)}e^{-\beta \mu } & =\frac{\varphi }{1-\varphi }\\ \beta J\varphi \left(k+1\right)-\beta \mu & =\log\;\varphi -\log\left(1-\varphi \right) \end{array}$$

Thus one retrieves the following formula for βμ:

(Equation s26) βμ=log(1−φ)−logφ+βJφ(k+1)

##### Calculating the spinodal-curve

As with the BP-model, the spinodal-curve can be determined by setting $\frac{\partial \left(\beta \mu \right)}{\partial \varphi }=0$, i.e.:

$$\begin{array}{ll} \frac{\partial \left(\beta \mu \right)}{\partial \varphi } & =-\frac{1}{1-\varphi }-\frac{1}{\varphi }+\beta J\left(k+1\right)=0\\ \beta J\left(k+1\right) & =\frac{1}{\varphi }+\frac{1}{1-\varphi }\\ \beta J\left(k+1\right) & =\frac{1}{\varphi \left(1-\varphi \right)} \end{array}$$

Thus one has for the spinodal-curve:

(Equation s27) $$\beta J=\frac{1}{\left(k+1\right)}\frac{1}{\varphi \left(1-\varphi \right)}$$

which is Equation 9 from the main text.

#### Calculating the binodal-curve

The binodal curve has been calculated through the Maxwell construction. We report an example here below in Figure S1 (BP for the binary system with K=3, β=3). This is obtained by the standard procedure of finding the point where the chemical potentials of the two phases are equal. Upon looking in Figure S1 (top) this geometrically corresponds to find the horizontal line whose intersection with the mean-field isothermal curve gives regions of equal area. We report here in Figure S1 (bottom) the binodal curves for the binary system with both approximations (CW and BP) alongside with the spinodals.

#### Finite size scaling

The systems we are considering here have short-range interactions and finite number of components (we are not dealing with a disordered systems) and thus, in regard to the study of the peak of specific heat as a function of the temperature, finite size effects shall follow established results of classical finite size scaling theory.^49^ In particular, in our case, the rounding and shifting of the transition point is ruled by terms of the order $O\left(L^{-d}\right)$ (L being the lattice size and d the dimension), as we show below in Figure S2

#### Calibration of the image processing algorithm

Figures 2 and 3 (main text) employ a novel image processing algorithm to detect phase separation in snapshots of numerical simulations. The algorithm is explained in the text and illustrated by the Figure 6 (main text). Here we elaborate on the algorithm and describe the required calibration procedure.

The image processing algorithm defines a measure of phase separation ξ, a continuous variable from 0 (well mixed) to 1 (phase separated). A threshold value needs to be calibrated based on comparison with numerical simulations of different systems along a simulated annealing pathway (β slowly increasing). Then, the method can be used for binary classification (mixed/separated). The Figure S3 shows the measure of phase separation ξ compared to the specific heat, as a part of the calibration procedure.

#### Further details on numerical simulations

In all simulations, the lattice was initialized randomly with a uniform distribution of all components. In case of the results presented in the Figure 2 (main text), the system was slowly evolved along a simulation pathway with the inverse thermodynamic temperature β increasing linearly from 0 to 8 over $10^{4}$ iterations (simulated annealing). During each iteration, the system was equilibrated for $10^{3}$ steps and the final snapshot was analysed for phase-separation. The total number of steps per system (defined by volume fraction φ) was therefore $10^{7}$, each step consisting of $L^{2}$ Kawasaki swaps, where L is the system size. For the Figure 3 (main text), the following procedure was employed. During an initial annealing period ($10^{4}$ steps), β was increased from 0 to 1, which was used for the rest of the simulation. Afterwards, the system was simulated for $10^{6}$ steps in order to reach equilibrium, which has been verified by observing a plateau in the measured total energy of the system. Finally, the simulation was advanced further $10^{6}$ steps during which a total of $10^{3}$ uniformly distributed snapshots were collected for present analysis.

#### Further details on the comparison between the cavity method results and numerical simulations

For sake of clarity, we report in Figure S4 phase diagrams for the ternary system where we superimpose the results from BP with the ones from numerical simulations: in all cases we are above 88% accuracy.

#### Experimental methods

##### General experimental conditions

Experiments were carried at room temperature in a 1536 well microplate (Greiner bio-one, item no.: 783096) where the coacervate forming components, i.e. Poly-L-lysine hydrochloride of length 10mer (Alamanda polymers, item: PLKC10) and Adenosine 5 ${}^{\prime }$-diphosphate sodium salt (Sigma Aldrich, cat. no.: A2754), were distributed, from 10X stock solutions, using Labcyte Echo 550 acoustic liquid dispenser. The phase diagram was obtained in 20mM Tris-HCl buffer at pH 9.0 which was dispensed using Fluidx XRD-384 reagent dispenser. After mixing the solutions, bright-field images of each of the wells were acquired using Yokogawa CellVoyager™ CV7000 high-throughput cytological discovery system at 60X magnification (Olympus objective UPLSAPO60XW, product no.: N1480800). The formation of coacervate was detected by visual inspection of the acquired images and by automated detection of phase separation (through image processing). All the chemicals were prepared in water at 200 mM concentrations. The buffer used here is Tris-HCl, which was prepared by dissolved 25 mM Trizma base (cat. no.: T1503) and was adjusted to pH 9.0 using hydrochloric acid.

##### Automated liquid handling

Automated liquid handling was performed using two different instruments. The Fluidx XRD-384 reagent dispenser was using to fill 3.2 μL of buffer solution in a 1536 well microplate (Greiner bio-one, item no.: 783096). The positive and the negative chemical species were dispensed using Labcyte Echo 550 acoustic liquid dispenser in a volume of 4 nL each. The remaining volume up to 4 μL was filled up with Milli-Q water. The .csv file that served as an input for the Labcyte Echo 550 was prepared using KNIME. The KNIME workflow takes an excel sheet with desired concentrations of positive and negative chemical species and performs required transformations in order to produce a .csv file that is Echo 550 readable. Once the dispensing is over the 1536 well microplate is vortexed in order to mix the two components. The microwell plate is then proceeded for microscopy. The Figure S5 provides a comprehensive view of the complete experimental pipeline.

## Data Availability

Source code for the parameter inference as well as the Monte Carlo numerical simulation is available online: https://github.com/ondrejtichacek/cavity-phase-sep.
