# Supplementary material for: Statistical mechanics of biomolecular condensates via cavity methods
Source: iScience. 2023 Mar 6;26(4):106300. doi: 10.1016/j.isci.2023.106300 (PMC10040705; doi:10.1016/j.isci.2023.106300)
Supplement: Document S1. Figures S1–S5 [file mmc1.pdf]

iScience, Volume *26*

Supplemental information

Statistical mechanics of biomolecular condensates via cavity methods

Nino Lauber, Ondrej Tichacek, Rudrarup Bose, Christoph Flamm, Luca Leuzzi, T-Y Dora Tang, Kepa Ruiz-Mirazo, and Daniele De Martino

Supplementary Figures

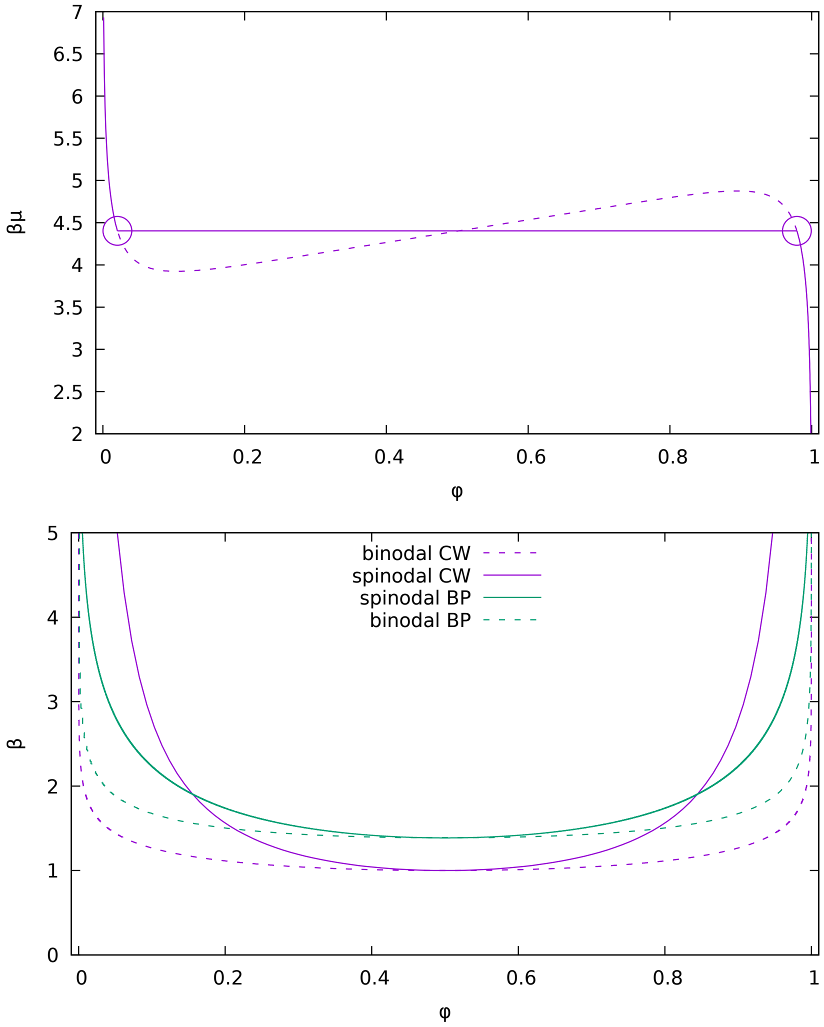

Figure S1: Mean field transition lines for the binary system.
Top: Maxwell construction for the isothermal curve $\beta\mu(\phi)$ for the binary system $K = 3$, $\beta = 2.2$ obtained by the cavity method (BP). Bottom: Phase diagram in the plane (ϕ, β) of the binary system (branching ratio $K = 3$) obtained via cavity method (BP, green) and regular solution model (CW, violet), both spinodal (continuous) and binodal (dashed) lines are depicted. Related to STAR Methods

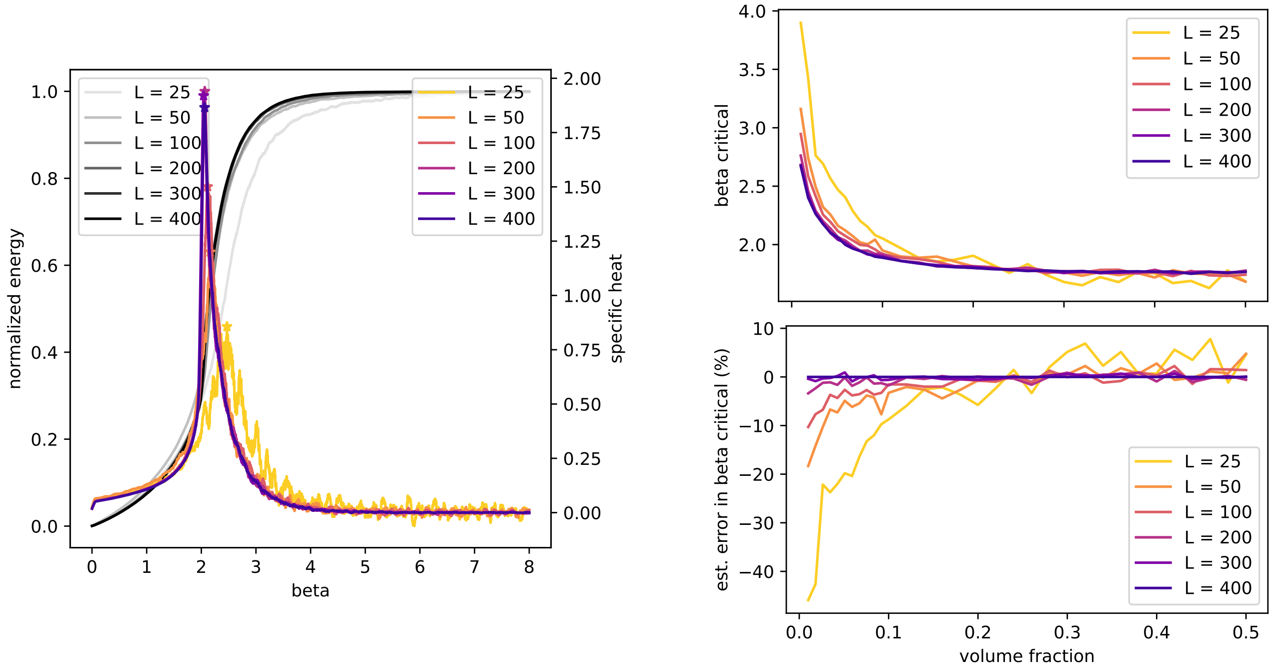

Figure S2: Effect of the system size L on the $\beta_{\mathbf{crit}}$.
Left: total normalized energy and the specific heat of the simulated systems (volume fraction = 0.05) for different lattice sizes. Right top: convergence of β_{crit} estimated from the simulation as the peak of the specific heat. Right bottom: estimated error of the β_{crit} with respect to the largest lattice size $L = 400$. The mean difference between $\beta_{\text{crit}}(L = 300)$ and $\beta_{\text{crit}}(L = 400)$ is less than 0.4 %. Related to STAR Methods

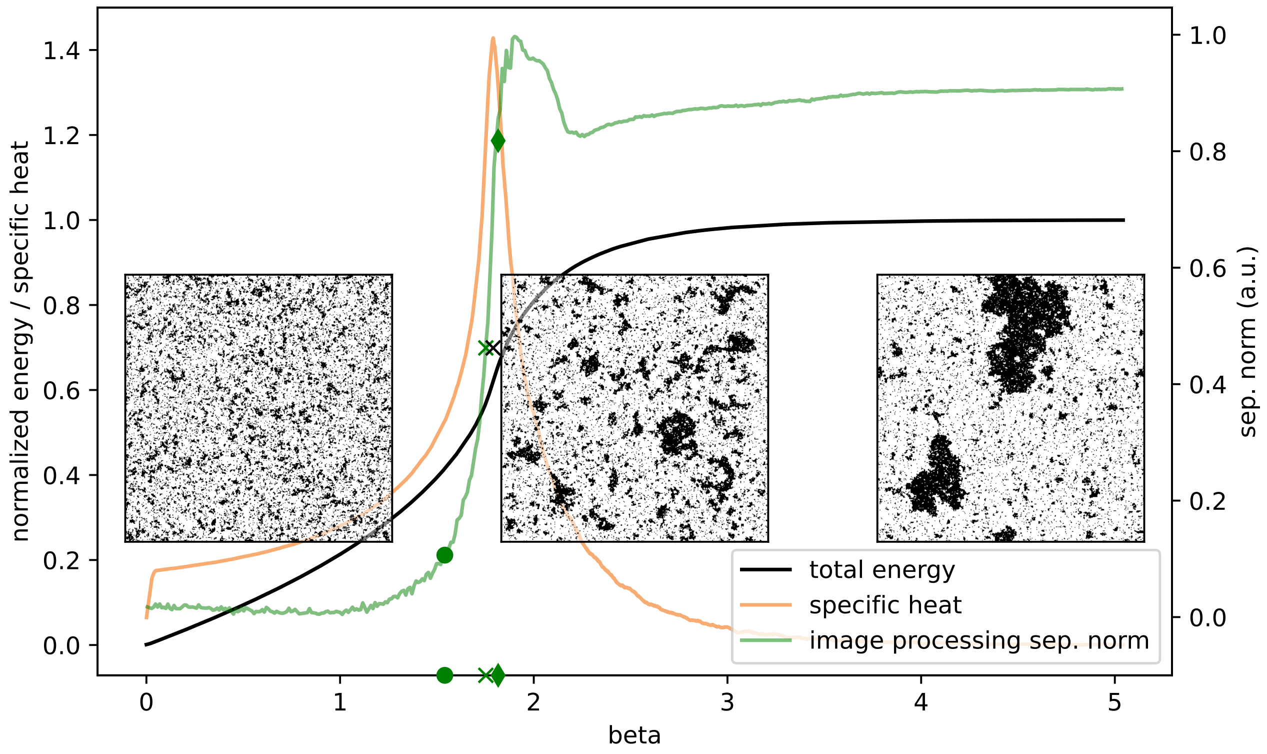

Figure S3: Calibration of the image processing algorithm.
Around the critical point β_{crit}, the measure ξ transitions continuously between 0 and 1. The value of 0.5 fits well with the location of the peak of the specific heat (see Fig. 2 in the main text). Systems where $\approx 0.1 < \xi < 0.5$ are in a meta-stable state. It turns out, that a threshold value of approx. 0.1 in turn corresponds well to the binodal curve of the BP mean field approximation. Three snapshots of the simulation show the system at different β corresponding to threshold $\xi = 0.1, 0.5, 0.9$ depicted in the graph by a green circle, cross, and diamond. Related to STAR Methods

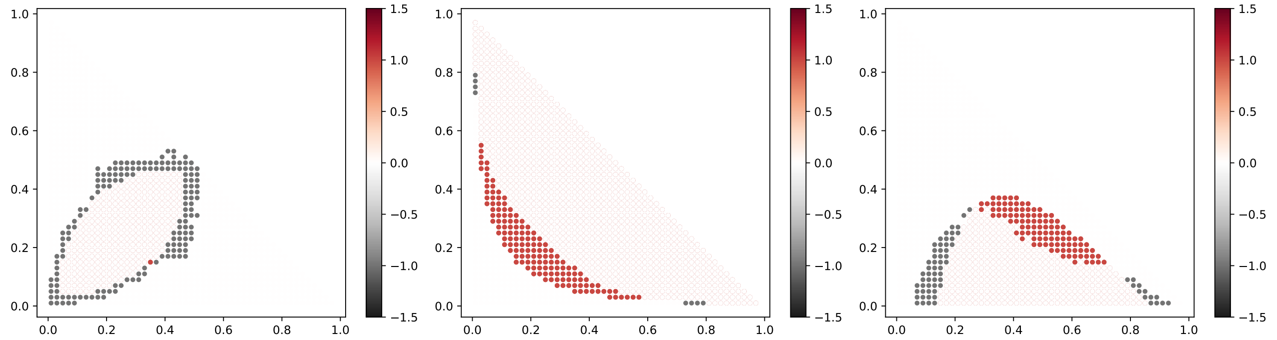

Figure S4: Phase diagrams for the ternary system, superimposed from BP and numerical simulations.

Gray points: mean field does not predict phase-separation where simulation does; red points: mean field predicts phase-separation where simulation does not. Related to STAR Methods

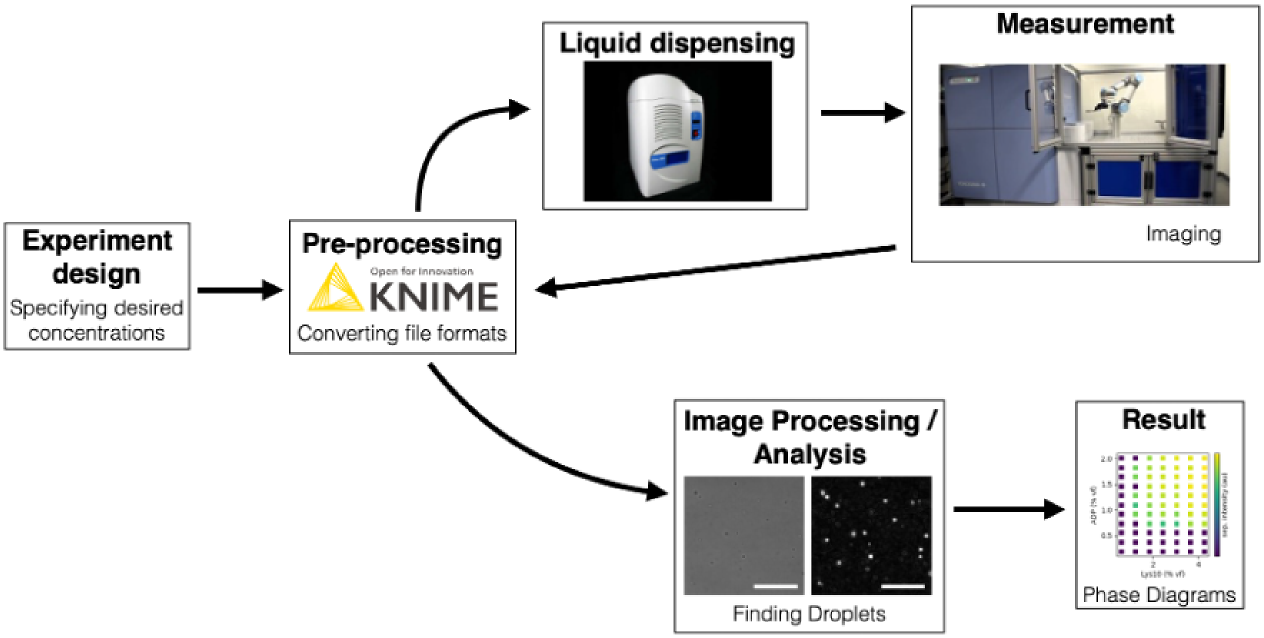

Figure S5: Overview of the experimental pipeline and subsequent data analysis. Related to STAR Methods.
